# Germline fate determination by a single ARGONAUTE protein in *Ectocarpus*

**DOI:** 10.1073/pnas.2518712123

**Published:** 2026-01-28

**Authors:** Viktoriia Bukhanets, Rita A. Batista, Fabian B. Haas, Rémy Luthringer, Jumana Kushkush, Min Zheng, Katharina Hipp, Vikram Alva, Claudia Martinho, Susana M. Coelho

**Affiliations:** ^a^Department of Algal Development and Evolution, Max Planck Institute for Biology, Tübingen 72076, Germany; ^b^Electron Microscopy Core Facility, Max Planck Institute for Biology, Tübingen 72076, Germany; ^c^Department of Protein Evolution, Max Planck Institute for Biology, Tübingen 72076, Germany; ^d^School of Life Sciences, Division of Plant Sciences, University of Dundee, James Hutton Institute, Dundee DD2 5DA, United Kingdom

**Keywords:** Argonaute, *Ectocarpus*, germline, sexual reproduction, mutant

## Abstract

ARGONAUTE (AGO) proteins are a highly conserved family of RNA-binding proteins that play central roles in gene regulation and developmental processes across eukaryotes. Although AGO family members have been extensively studied in animals and plants, where they are typically encoded by multiple genes, their function in brown algae, a diverse and complex group of multicellular algae, remains largely unknown. Here, we show that the genomes of several brown algae encode only a single AGO protein, containing the conserved functional domains characteristic of the family. Using the model brown alga *Ectocarpus* and a combination of cell biology, genetic, and transcriptomic approaches, we demonstrate that AGO is essential for the transition from vegetative growth to sexual reproductive development and for germline establishment. Our results further suggest that AGO functions in concert with microRNAs to regulate target genes primarily at the posttranscriptional level, likely through translational repression. *Ectocarpus* thus represents a rare example of a complex multicellular organism that relies on a single AGO protein to regulate key developmental processes, pointing to a minimalistic model of RNA-based regulation in brown algae.

## Significance

ARGONAUTE (AGO) proteins are central regulators of gene expression in multicellular eukaryotes, mediating developmental processes through interactions with small RNAs. While extensively studied in plants and animals, the diversity of AGO function across independently evolved multicellular lineages remains poorly understood. Using brown algae as a comparative model, we demonstrate that a single AGO protein can direct key developmental transitions and germline specification likely through posttranscriptional regulation. These findings reveal a minimalist yet effective RNA silencing system and provide insights into the evolutionary versatility of AGO proteins in coordinating complex developmental programs across eukaryotes.

ARGONAUTE (AGO) proteins are a highly conserved family of nucleic acid–binding proteins essential for RNA-guided gene regulation. They bind a variety of small RNAs (sRNAs) and guide them to complementary nucleic acid targets to mediate transcriptional or posttranscriptional gene silencing (1–3). AGO proteins regulate gene expression to mediate antiviral response and suppress transposable element activity (4, 5). They are found across all major eukaryotic lineages and in some prokaryotes, where they are also thought to function in defense processes, suggesting a deeply conserved evolutionary role in nucleic acid–based immunity (4, 6, 7). AGO proteins are also known to regulate development and stress responses (8, 9), playing essential roles in processes such as embryogenesis (10), organ formation (11), and pathogen responses (12).

In eukaryotes, AGO proteins fall into two major clades: AGO and PIWI (1–3). AGO-clade proteins typically associate with 21–22 nt microRNAs (miRNAs) or 21–24 nt small interfering RNAs (siRNAs), which mediate posttranscriptional gene silencing (PTGS) through mRNA cleavage, translational repression, or RNA decay (13–15). These sRNAs are typically processed from double-stranded RNA precursors by Dicer enzymes, generating duplexes with 2-nt 3′ overhangs. One strand of the duplex is selectively loaded into an AGO protein, forming the core of the RNA-induced silencing complex (RISC). The guide RNA then directs RISC to complementary mRNA targets via base pairing, leading to target cleavage, translation repression, or mRNA decay (13–15).

PIWI-clade proteins, in contrast, associate primarily with 21–35 nt PIWI-interacting RNAs (piRNAs) in animals, which are derived from single-stranded precursors processed independently of Dicer proteins (16). piRNAs guide PIWI proteins to transposable element transcripts in the germline, promoting their degradation or transcriptional silencing through chromatin modifications and deposition of repressive histone marks (17). Notably, plants lack PIWI proteins but have functionally diversified AGO-clade members (9, 18): while some plant AGOs (e.g., AGO1) mediate PTGS using miRNAs (19), others (e.g., AGO4) fulfill PIWI-like functions in transcriptional gene silencing (TGS) via the RNA-directed DNA methylation (RdDM) pathway (20, 21). These AGOs bind 24-nt siRNAs and direct DNA methylation at homologous loci, particularly transposons and repeats, establishing epigenetic silencing (20, 21).

Despite their broad distribution and functional importance, AGO proteins remain poorly characterized in some eukaryotic lineages. One such understudied group is the brown algae (Phaeophyceae), a lineage of multicellular photosynthetic Stramenopiles, distinct from animals and land plants, that independently evolved complex multicellularity ~450 Mya (22). Although at least one AGO has been identified in the model filamentous brown alga *Ectocarpus* (23), its function and conservation across other brown algae remain unexplored.

Brown algae exhibit remarkable morphological and developmental diversity, from simple filamentous forms to complex thallus structures rivaling those of plants (22). Many brown algae, including kelps, form extensive underwater forests that provide critical marine habitats and contribute significantly to global carbon fixation (22). Most brown algae follow a haploid-diploid life cycle, alternating between a diploid sporophyte and a haploid gametophyte, each with distinct morphology and developmental programs (24).

**Fig. 1.**
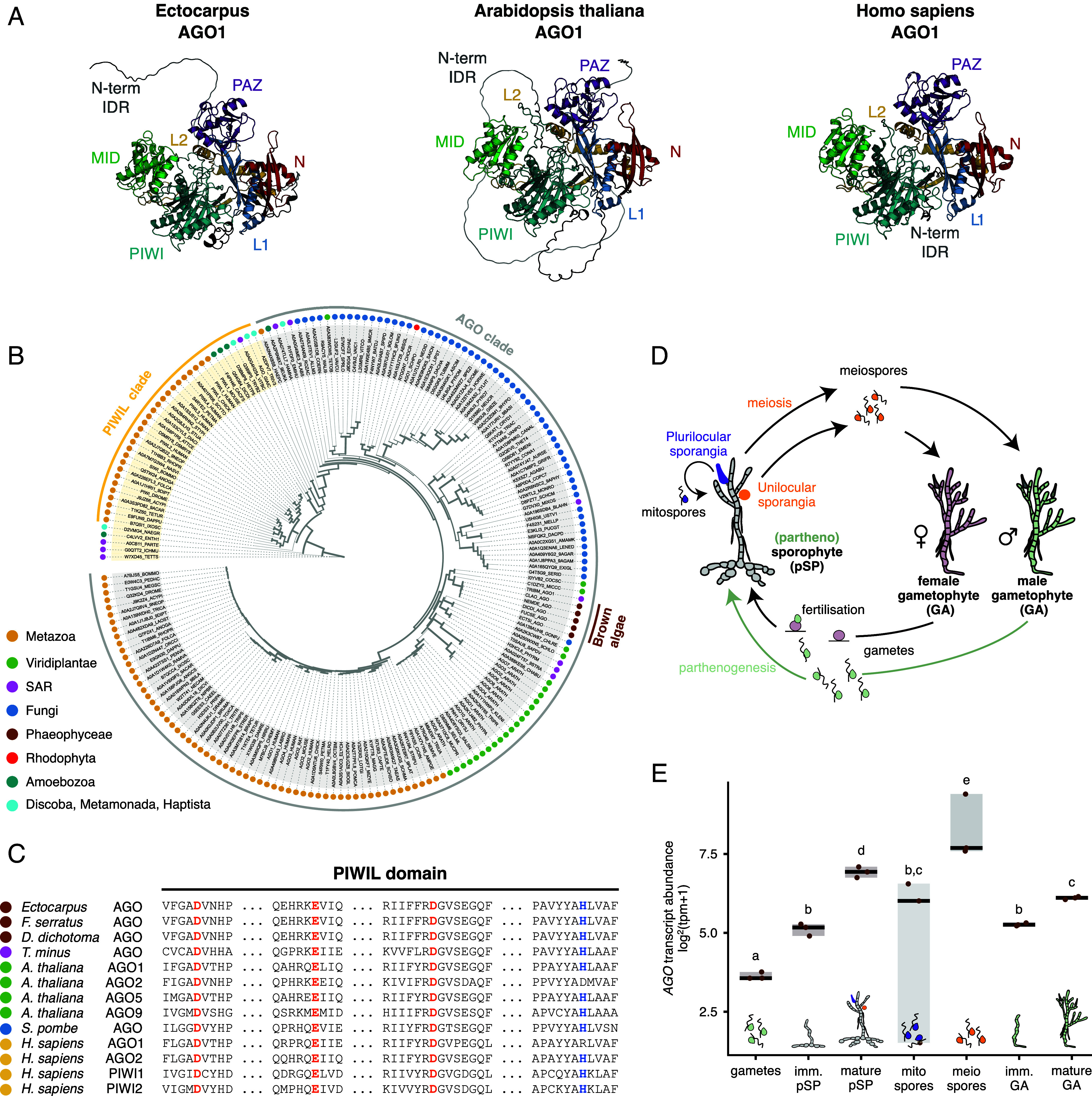
Structural and evolutionary insights into brown algal ARGONAUTE proteins. (*A*) AlphaFold3-predicted structures of representative ARGONAUTE proteins from *Ectocarpus* sp. (*Left*), *Arabidopsis thaliana* (*Middle*), and *Homo sapiens* (*Right*) shown in cartoon representation. Each structure is color‐coded by domain: N‐terminal (red), L1 (blue), PAZ (purple), L2 (gold), MID (green), and PIWI (teal). Unstructured N‐terminal regions (IDRs) are indicated in light gray. (*B*) Phylogenetic tree illustrating the distribution of Argonaute (AGO) and PIWIL clades across major eukaryotic lineages, with each tip representing an AGO/PIWIL sequence. Outer dots indicate taxonomic groups: Metazoa (gold), Viridiplantae (green), SAR (magenta), Fungi (blue), Phaeophyceae (brown), Rhodophyta (red), Amoebozoa (teal), and Discoba/Metamonada/Haptista (cyan). The PIWIL clade is on the left side of the circle, and the AGO clade is on the right. Branch thickness reflects SH-like aLRT support values, which estimate the statistical confidence of each split based on local likelihood ratio tests. (*C*) The conservation of the catalytic tetrad DEDH motif in the PIWIL domain is shown in a partial multiple sequence alignment of brown algal AGO proteins (*Ectocarpus*, *Fucus serratus*, *Dictyota dichotoma*) and *Tribonema minus*, alongside sequences from *A. thaliana*, *Schizosaccharomyces pombe*, and *H. sapiens*. The signature DEDH catalytic tetrad is highlighted in bold. Dots represent omitted regions for clarity. (*D*) Life cycle of *Ectocarpus* has both sexual and asexual phases. In the sexual cycle, female and male haploid gametophytes, carrying a U or V sex chromosome respectively, produce gametes that fuse to form a diploid zygote, which then develops into a diploid sporophyte. The sporophyte releases haploid meiospores via meiosis, which germinate into new gametophytes. In the asexual cycle, the diploid sporophyte can also produce diploid spores through mitosis, which develop into new sporophytes without gamete fusion, ensuring rapid reproduction. Unfertilized gametes can also go into an asexual cycle by growing into haploid partheno-sporophytes which are indistinguishable from diploid sporophytes. GA: gametophyte; pSP: partheno-sporophyte. (*E*) Transcript abundance of AGO during the life cycle of *Ectocarpus*. Different letters above the plots indicate significant differences (*P* < 0.05, Kruskal–Wallis test with Bonferroni correction).

In *Ectocarpus*, recent advances in molecular and genomic tools have begun to elucidate the regulation of these transitions (25–27). In particular, conserved TALE homeodomain transcription factors and dynamic histone modifications regulate the gametophyte-to-sporophyte transition (28–31). The reverse transition—from sporophyte to gametophyte—occurs through meiosis (or apomeiosis) (32, 33), during which meiospores are reprogrammed to initiate the algal germline (i.e., the gametophyte developmental program), ultimately producing gametes (Fig. 1*D*) (24). Unlike animals, which typically specify the germline early in embryogenesis, brown algae, like plants, lack an early-segregated germline (34–36), and the molecular mechanisms governing meiospore reprogramming remain largely unknown, raising the question of whether sRNA pathways and AGO proteins may play roles in this developmental transition, as they do in animals and plants (37–41).

Here, we investigate the evolution and function of AGO proteins in brown algae. We first characterize their domain features and phylogenetic relationships, showing that brown algae possess a single, highly conserved AGO more closely related to plant AGOs than to animal AGOs. Using the model *Ectocarpus*, we employed reverse genetics and transcriptomic profiling to dissect the biological role of AGO. We show that *AGO* is specifically upregulated during the sporophyte-to-gametophyte transition, coinciding with the onset of germline specification. While *ago* knockout mutants develop normally as sporophytes and undergo meiosis, most mutant meiospores fail to develop, and those that do mainly adopt a sporophyte rather than a gametophyte fate. Our findings suggest that AGO is essential for the establishment of the gametophyte fate in a non-cell-autonomous manner during meiospore development. Furthermore, sRNA sequencing confirmed that most *Ectocarpus* sRNAs are 21-nt in length, and that a subset of these, particularly those potentially originating from miRNAs, associate with AGO. Altogether, our results indicate that a single multifunctional AGO orchestrates life cycle transitions via sRNA- and miRNA-mediated regulation of target loci, highlighting the evolutionary versatility and central developmental role of AGO in brown algae.

## Results

### A single ARGONAUTE protein in *Ectocarpus*.

While most prokaryotic species typically harbor a single AGO homolog, eukaryotes often possess an expanded set, apart from a few notable exceptions such as the unicellular organisms *S. pombe* and *Giardia* (42). This expansion is particularly pronounced in multicellular eukaryotes: for instance, *A. thaliana* encodes 10 *AGO* genes (18), whereas *H. sapiens* carries eight (1). The increased number of AGO homologs in plants and animals likely reflects more complex regulatory needs, including the diverse roles AGOs fulfill in mediating RNA interference (RNAi) and other gene-silencing mechanisms. Given this context, it is surprising that *Ectocarpus*, a complex multicellular brown alga, appears to deviate from this pattern by harboring only a single AGO homolog (23). To check for highly divergent homologs that might have been overlooked, we carried out a proteome-wide search of *Ectocarpus* using the sensitive sequence comparison method HHsearch (43). Our analysis did not reveal any additional candidates, confirming the presence of a single AGO in this species (Fig. 1). Moreover, when examining other publicly available brown algal genomes and transcriptomes, including *Fucus serratus*, *Fucus distichus*, *Dictyota dichotoma*, *Nemacystus decipiens*, and *Cladosiphon okamuranus,* we consistently found only one AGO homolog per species, suggesting that this may be a distinctive feature of brown algae (Fig. 1). To position brown algal AGOs more precisely within the eukaryotic lineage, we constructed a phylogenetic tree using representative AGO and PIWI-like protein (PIWIL) sequences from across the eukaryotic domain. In the resulting tree, AGO proteins cluster into two principal clades: “PIWIL” and “AGO” (Fig. 1B). Brown algal AGOs clearly segregate into the AGO clade, grouping with other Stramenopile and plant AGOs. Consistent with previous studies (44, 45), plant and metazoan AGOs cluster together rather than forming the expected fungal-metazoan clade of Opisthokonta, a topology likely shaped by uneven evolutionary rates and independent lineage-specific expansions in plants and metazoans, together with pronounced divergence and recurrent loss of AGOs in fungi.

Like other AGO family proteins, brown algal AGOs consist of four globular domains (N, PAZ, MID, and PIWI), connected by two short linker regions (L1 and L2) (Fig. 1*A* and *SI Appendix*, Fig. S1). Additionally, the N-terminal domain is preceded by an intrinsically disordered region (IDR) of approximately 125 residues. While brown algal AGOs share 82 to 92% sequence identity among themselves, the N terminus is more divergent than the rest of the protein. Crucially, the canonical DEDH tetrad in the PIWI domain—a motif essential for RNA slicing (endonuclease) activity—remains fully conserved across all brown algal AGOs (Fig. 1*C* and *SI Appendix*, Fig. S2). In BLAST searches, the closest sequence matches to brown algal AGOs are the two AGO homologs of *T. minus*, a member of the sister group Xanthophyceae within the Stramenopiles clade, sharing ~45% sequence identity. The next-best matches, AGO homologs from fungi and plants, display 43 to 44% identity, while metazoan AGOs (e.g., those from *H. sapiens*) share roughly 40% (*SI Appendix*, Fig. S2). The Stramenopile lineage as a whole typically encodes only one or two AGO homologs, in contrast to the multiple AGOs seen in plants and animals. Among the 10 AGO proteins in *A. thaliana*, brown algal AGOs show the highest sequence similarity to AGO1, AGO5, and AGO10, indicating potential roles in posttranscriptional gene silencing through interactions with miRNAs and/or siRNAs (Fig. 1*A* and *SI Appendix*, Fig. S1) (19). Notably, despite the high degree of overall sequence and structural similarity to other eukaryotic AGOs, the N-terminal IDR in brown algal AGOs is divergent in sequence compared to other eukaryotic AGOs.

To initially assess whether the *Ectocarpus* AGO might be functional, we examined *AGO* expression across different developmental stages. The life cycle of *Ectocarpus* involves an alternation between a gametophyte and a sporophyte generation (Fig. 1*D*) (29). When asexual reproduction takes place, haploid partheno-sporophytes are formed, and these are indistinguishable from diploid sporophytes in terms of developmental patterns and morphology. The (partheno-)sporophyte produces approximately 100 haploid meiospores through meiosis, followed by multiple mitotic divisions, which are stored within unilocular sporangia (46). In *Ectocarpus*, *AGO* transcripts accumulate in mature partheno-sporophytes bearing fully developed unilocular sporangia, as well as in meiospores produced and released from these structures (Fig. 1*E* and Dataset S1). This expression pattern suggests a potential role for AGO in mediating the transition between the vegetative growth phase of the partheno-sporophyte and the establishment of the gametophytic germline generation.

### AGO Is Required for the Transition to the Gametophyte Generation.

**Fig. 2.**
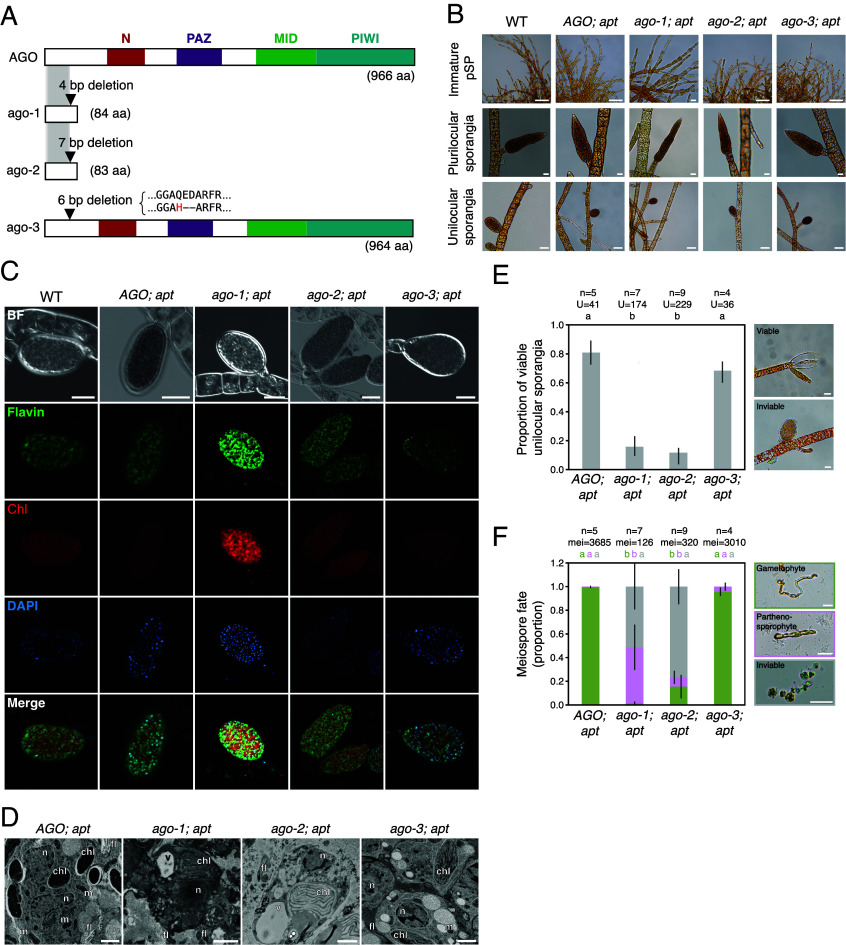
Phenotypic characterization of *Ectocarpus* ago mutants. (*A*) Schematic representation of the wild-type (WT) *Ectocarpus* AGO protein and the predicted truncated proteins of each of the CRISPR-generated ago mutants. The total predicted length of each protein is indicated in parenthesis. (*B*) Bright-field images WT, *AGO; apt* and *ago* mutant partheno-sporophytes, with their respective plurilocular and unilocular sporangia. (Scale bars, 50 µm.) (*C*) Epifluorescence microscopy images of WT, *AGO; apt* and *ago* mutant unilocular sporangia, showing different channels: BF (bright-field), Flavin (autofluorescence of the flavin protein present in the posterior flagella of the meiospores), Chl (chlorophyl autofluorescence), DAPI, and Merge (composite of Flavin, Chl, and DAPI channels). (Scale bars, 20 µm.) (*D*) Transmission electron micrographs of *AGO; apt* and *ago* mutant unilocular sporangia. Chl: chloroplast, n: nucleus, v: vacuole, fl: flagellum, m: mitochondrium. (Scale bars, 1 µm.) (*E*) Viability of unilocular sporangia of ago mutant and control *AGO; apt* algae. n corresponds to the total number of analyzed partheno-sporophytes, U corresponds to the total number of analyzed unilocular sporangia. Different letters above the plots indicate significant differences (*P* < 0.05, Kruskal–Wallis test with Bonferroni correction). (Scale bars, 20 µm.) (*F*) Developmental fate of meiospores in *ago* mutants and *AGO; apt* controls. In WT and *AGO; apt*, meiospores develop into gametophytes (green box, see also Fig. 1*D*). In *ago-1; apt* and *ago-2; apt*, many spores are inviable (gray box) or develop into partheno-sporophytes (pink box). Different letters above the plots indicate significant differences (*P* < 0.05, Kruskal–Wallis test with Bonferroni correction). (Scale bars, 20 µm.)

To test this hypothesis, we employed CRISPR/Cas (47, 48) to generate *AGO* (Ec-01_00900) knockout (KO) lines in haploid partheno-sporophytes, where mutations are directly expressed phenotypically in a haploid state, facilitating mutant screens (29). CRISPR/Cas resulted in three independent *ago* mutant lines that also contain an additional mutation at the *APT* locus, a selective gene which allows for screening of mutants (see *Materials and Methods* for more details). As a control, a line exclusively mutated for *APT* was also generated, since it has been previously shown that mutations in *APT* alone induce significant transcriptional, but no phenotypic changes (48). These mutant lines are hereafter referred to as *ago-1; apt, ago-2; apt, ago-3; apt, and AGO; apt* (Fig. 2*A* and Dataset S2). *ago-1; apt* has a 4 bp deletion in the first exon of the *AGO* gene. Similarly, *ago-2; apt* has a 7 bp deletion, and *ago-3; apt* has an in-frame deletion of 6 bp in the same exon (Fig. 2*A* and *SI Appendix*, Fig. S3). The mutations in *ago-1; apt* and *ago-2; apt* lead to a premature stop codon and a significantly disrupted predicted protein. In contrast, *ago-3; apt* carries a missense mutation (Q69H), followed by a 2 amino acid deletion, but the structure and function of the predicted protein is expected to remain largely unaffected relative to wild-type (Fig. 2*A*). Therefore, *ago-3; apt* functions as an effective silent mutant control to study the effects of AGO loss.

We examined the phenotype of the *ago-1; apt*, *ago-2; apt* and *ago-3; apt* mutants in comparison to that of control lines (wild-type, and *AGO; apt)* across the life cycle of *Ectocarpus* and found no evidence for

distinctive modifications in the morphology of the *ago* partheno-sporophytes (Fig. 2*B*). Similarly to wild-type and AGO; *apt*, *ago* mutant partheno-sporophytes produced plurilocular sporangia (where asexual spores are produced by mitosis, see Fig. 1*D*), and the new partheno-sporophytes developed upon germination of these asexual mitospores were indistinguishable to wild-type partheno-sporophytes (Fig. 2*B*).

Given that the transcriptional profile of *AGO* during the life cycle of *Ectocarpus* indicates high expression in mature partheno-sporophytes and meiospores—the transition between vegetative and reproductive gametophyte development (Fig. 1*D*)—we sought to determine whether AGO is indeed required for this transition. To test this, we incubated *ago* mutant and control partheno-sporophytes under conditions that induce the formation of unilocular sporangia and examined these structures for any possible developmental or morphological changes. All *ago* mutant partheno-sporophytes produced unilocular sporangia, and these structures were indistinguishable from wild-type in terms of morphology using light microscopy (Fig. 2*B*). We also examined the unilocular sporangia with epifluorescence microscopy but failed to detect any differences between *ago* mutants and control (Fig. 2*C*). Closer inspection with transmission electron microscopy (Fig. 2*D*) also revealed no difference in the ultrastructure of the meiospores. Therefore, we conclude that AGO is not required for the initiation of unilocular sporangia in partheno-sporophytes, nor for meiosis (and further mitosis) leading to the formation of meiospores.

However, we observed a striking difference between the *AGO;* *apt* and *ago-1; apt* or *ago-2; apt* mutant lines when these unilocular sporangia became mature. Specifically, more than 80% of the *ago-1; apt* and *ago-2; apt* unilocular sporangia aborted at maturity (i.e., did not release their meiospores and died) compared to around 20%-30% in both *AGO; apt* and *ago-3; apt* (Fig. 2*E* and Dataset S4).

In wild type *Ectocarpus* healthy meiospores are released from unilocular sporangia and develop into gametophytes, representing a successful transition from the sporophytic to the sexual reproductive generation ((29, 49), Fig. 1*D*). Gametophytes are easily recognizable due to their typical morphology (26, 29, 50): following germination, the first cell division is asymmetrical leading to the production of both an upright and a rhizoid cell which has a wavy morphology (Fig. 2*F*). In the control lines *AGO; apt* and *ago-3; apt*, nearly all meiospores released from unilocular sporangia developed into gametophytes, as expected (Fig. 2*F*). In contrast, a large proportion of meiospores released from *ago-1; apt* and *ago-2; apt* unilocular sporangia (approximately 50% and 75%, respectively) were inviable and died within 24 h (Fig. 2*F*). Among the surviving spores, a significant fraction developed into partheno-sporophytes rather than gametophytes: 47% in *ago-1; apt* and 8% in *ago-2; apt* lines (Fig. 2*F*). Together, these observations indicate that while AGO is dispensable for the development of vegetative partheno-sporophytes and for the asexual life cycle, it is required for the production of viable meiospores and for the successful transition into the gametophyte generation.

### AGO Affects the Gametophytic Transition Through a Sporophytic Non-Cell-Autonomous Mechanism.

Although the vast majority of *ago* meiospores formed in haploid male partheno-sporophytes are inviable or develop into the incorrect generation, we successfully isolated a single *ago-1; apt* mutant individual that displayed a developmental pattern resembling that of a gametophyte. Upon maturation, this individual produced functional gametes, confirming it was a bona fide gametophyte. Male gametes from this mutant were crossed with gametes of a wild type female strain (Ec25), yielding a heterozygous diploid sporophyte (*AGO/ago-1; APT/apt*) (*SI Appendix*, Fig. S4 and Dataset S5). This allowed us to test whether the defects in meiospore viability and identity observed in haploid *ago* mutants were due to a cell-autonomous effect in the meiospores themselves or to a non-cell-autonomous effect originating from the surrounding parental sporophyte cells.

The isolated heterozygous *AGO/ago-1; APT/apt* sporophyte exhibited a wild-type phenotype and produced normal unilocular sporangia at maturity, which we microdissected in order to analyze the developmental fate of their meiospores. The resulting meiospores were viable and all developed into healthy gametophytes. Genotyping revealed that 21 gametophytes carried the *ago-1* mutant allele, while 17 carried the wild-type *AGO* allele, consistent with the expected Mendelian segregation for a single-locus mutation (χ^2^ test, *P* = 0.516) (Table 1). These findings, together with our previous observations, indicate that the *ago* mutant phenotype manifests only in a haploid mutant context. In heterozygous diploid sporophytes, the *ago* mutation does not affect meiospore development, as meiospores remain viable regardless of whether they carry a functional *AGO* allele or a mutated one. This suggests that *AGO* functions through a sporophyte-derived or parental carry-over mechanism, acting in a non-cell-autonomous manner to regulate meiospore viability and fate.

**Table 1.** Gametophyte progeny of *APT/apt AGO/ago-1* sporophytes

	Observed count	Expected	χ^2^=(O − E)2/E	df	Critical χ^2^ (α = 0.05)
***apt***	10	10	0.000	1	3,841
***APT***	10	10	0.000		
				χ^2^ = 0.000, *P* = 1.000	
***ago-1***	21	19	0.211	1	3,841
***AGO***	17	19	0.211		
				χ^2^ = 0.421, *P* = 0.516	

### AGO Mutations Do Not Substantially Affect Gene Transcript Abundance.

**Fig. 3.**
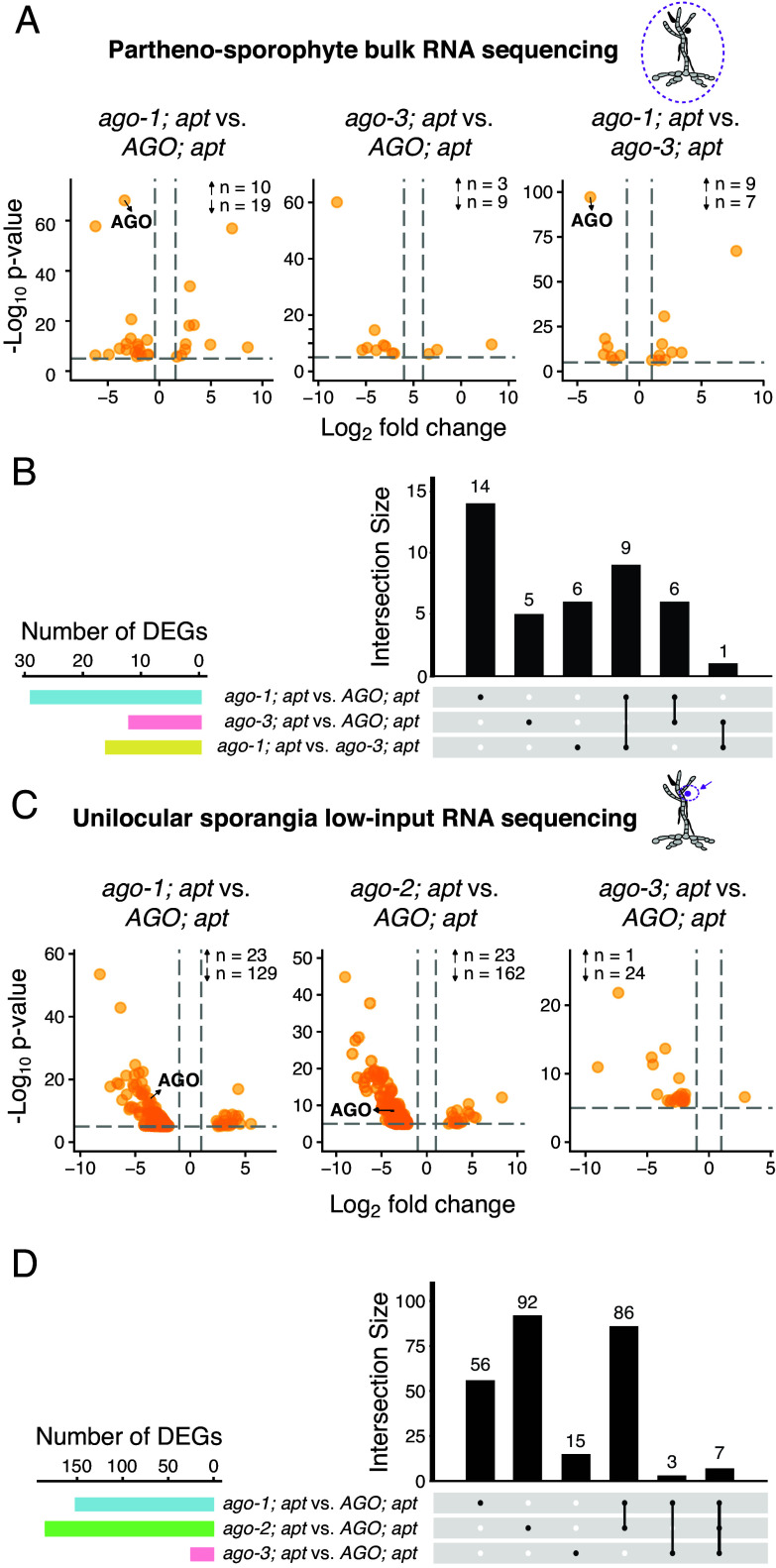
Transcriptomic analysis of *ago* mutants using bulk partheno-sporophyte and low-input unilocular sporangia mRNA sequencing. (*A*) DEGs identified by bulk mRNA sequencing of mature partheno-sporophytes. Strain comparisons are indicated above each plot. n refers to the number of upregulated (↑) and downregulated (↓) DEGs in each comparison. Only DEGs are shown. (*B*) UpSet plot showing the overlap of DEGs from the comparisons in panel *A*. (*C*) DEGs identified by low-input mRNA sequencing of microdissected unilocular sporangia. Strain comparisons are indicated above each plot. n refers to the number of upregulated (↑) and downregulated (↓) DEGs in each comparison. Only DEGs are shown. (*D*) UpSet plot showing the overlap of DEGs from the comparisons in panel *C*. Further strain comparisons are represented in *SI Appendix*, Fig. S5. Detailed DEG lists can be found in Datasets S6 and S7.

Given the similarity between the *Ectocarpus* AGO and the *A. thaliana* AGO1, which is known to mediate PTGS via miRNA-guided mRNA cleavage and degradation (19), we hypothesized that *Ectocarpus* AGO might function in a similar manner. To investigate this, we performed bulk RNA sequencing of mature *ago-1; apt* partheno-sporophytes, along with the respective *ago-3; apt* silent mutant and *AGO; apt* controls (Fig. 3 *A* and *B*). Comparative transcriptomic analysis revealed only 29 differentially expressed genes (DEGs) between *ago-1; apt* and *AGO; apt*, the majority of which were downregulated in the mutant. Similarly, we found 16 DEGs between the silent mutant *ago-3; apt* and the *AGO; apt* control (Fig. 3*A*). Additionally, only 14 DEGs were exclusive of the *ago-1; apt* and not present in the silent mutant *ago-3; apt* (Fig. 3*B*). Functional annotation of these DEGs did not reveal an enrichment for any specific biological processes or pathways (Dataset S6). *AGO* itself was strongly downregulated in the *ago-1; apt* mutant (Fig. 3*A*), consistent with possible degradation of the altered transcripts. Overall, these bulk RNA-seq results show no major transcriptional changes in *ago* mutants, in line with the localized phenotypic differences observed in unilocular sporangia and meiospores (Fig. 2 *E* and *F*).

In order to identify potential transcriptional changes specific to this tissue, we performed low-input RNA sequencing on microdissected unilocular sporangia (see *Materials and Methods* for more details). This analysis identified 152 and 185 DEGs in *ago-1; apt* and *ago-2; apt*, respectively, when compared to the *AGO; apt* control, with the majority of DEGs being downregulated in the mutants. Of these, 93 DEGs were shared between the two mutant backgrounds (Fig. 3 *C* and *D* and *SI Appendix*, Fig. S5 and Dataset S7). Additionally, in both bulk and low-input RNA-seq experiments, the large majority of identified DEGs corresponds to protein-coding genes, with only a few transposable elements (TEs) showing altered expression (Datasets S6 and S7).

Collectively, these results indicate that *ago* mutations in *Ectocarpus* lead to relatively limited changes in transcript abundance. The predominance of downregulated DEGs contrasts with the expected outcome of canonical *A. thaliana* AGO1-like activity, which typically results in miRNA-guided transcript cleavage and degradation. This discrepancy suggests that the small number of observed transcriptional changes may be indirect or, alternatively, that AGO might contribute to increase mRNA production and/or stability (51). *Ectocarpus* AGO may therefore function via mechanisms such as transcriptional regulation or translational repression, rather than through widespread cleavage-based PTGS, although it is not possible to completely exclude a scenario wherein cleaved transcripts would still accumulate and be detected in our RNA-sequencing experiments.

### AGO Associates with 21-nt sRNAs.

To further investigate the role of AGO in *Ectocarpus* and its association with sRNA molecules, we performed total sRNA sequencing in partheno-sporophytes of the *ago-1; apt* mutant, the *ago-3; apt* silent mutant, and the *AGO; apt* control line. In parallel, we applied the Trans-kingdom, Rapid, Affordable Purification of RISCs (TraPR) protocol (52) to *AGO; apt* control partheno-sporophytes to identify RISC-bound sRNAs.

**Fig. 4.**
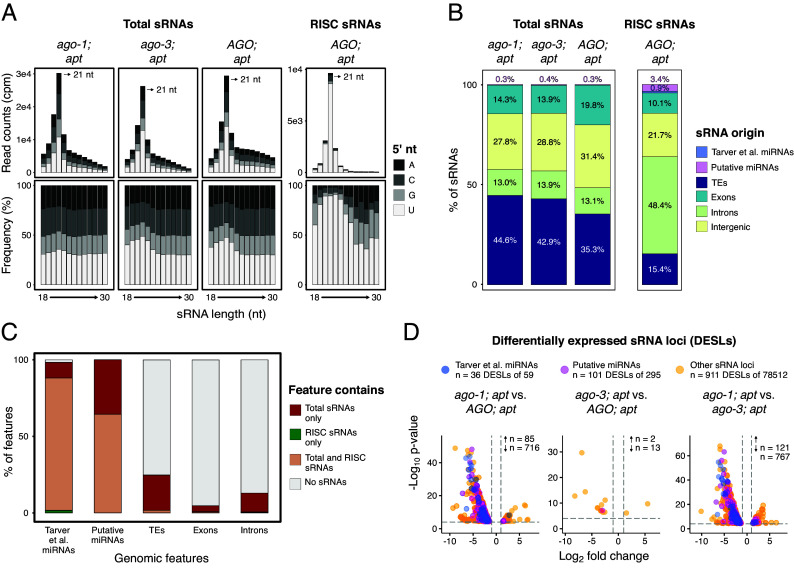
Characterization of total and RISC-associated sRNAs in *ago* mutants and control strains. (*A*) Abundance, size distribution, and 5′ nucleotide composition of total and RISC-associated sRNAs in different *ago* mutants and the *AGO; apt* control. RISC-associated sRNAs were isolated using the TraPR system. (*B*) Genomic origin of total and RISC sRNAs in *ago* mutants and the *AGO; apt* control. sRNAs sequences were classified based on the genomic regions they map to, as indicated by the color code. (*C*) Presence of sRNAs in selected genomic features. Colors indicate whether each feature contains only total sRNAs, only RISC sRNAs, both, or none. (*D*) Differentially expressed sRNA loci (DESLs) identified from total sRNA sequencing. The *Top* panel shows the total number of loci derived from Tarver et al. and putative miRNAs, with the number of differentially expressed loci specified for each category. Strain comparisons are indicated above each plot. n corresponds to the number of upregulated (↑) and downregulated (↓) DESLs in each comparison. Only DESLs are shown in this plot.

As previously reported by (23), most sRNAs detected in *Ectocarpus* are 20–22 nucleotides (nt) long, with a strong predominance of 21-nt sRNAs (Fig. 4A). Across all mutant and control samples, 21-nt sRNAs show a strong 5′-uridine (5′U) bias. This pattern becomes even more pronounced when considering only RISC-bound sRNAs (Fig. 4*A*, RISC sRNA panel), where approximately 90% of 21-nt sRNAs begin with a 5′U, consistent with the known preference of AGO1-clade proteins for these sRNAs and suggesting the same happens in *Ectocarpu*s AGO (18).

To determine the origin of these sRNAs, we assessed the proportion of reads mapping to TEs, exons, introns, intergenic regions, and miRNA genes (Fig. 4*B*). We used the previously published miRNA loci annotation from Tarver et al. (23) and supplemented it with our own set of putative miRNAs loci, which we were able to identify and curate due to the high-coverage sRNA sequencing dataset generated in this study.

Putative miRNA gene were defined as sequences derived from inverted repeat regions that produce both mature miRNA and miRNA* molecules, with a predominance of putative mature miRNA reads (see *Materials and Methods* for details). In total, of the 354 miRNAs used in this study, 59 were previously reported by Tarver et al. (23) (Dataset S8), and 295 were newly identified putative miRNAs (Dataset S9). In the *AGO; apt* control sample, approximately one third of sRNAs originated from TEs, and another third from intergenic regions (Fig. 4*B*), consistent with previous observations by Tarver et al. (23). In both *ago-1; apt* and *ago-3; apt* lines, the proportion of TE-derived sRNAs was slightly increased relative to the *AGO; apt* control (Fig. 4*B*), and this was not accompanied by changes in sRNA size distribution (Fig. 4*A*). Across these three samples, only around 0.3 to 0.4% of sRNAs mapped to annotated miRNA genes, reflecting the relatively low abundance of this genomic feature. In contrast, the pool of RISC-bound sRNAs obtained through the TraPR protocol showed a dramatic shift: TE-derived sRNAs were strongly depleted, while sRNAs mapping to introns and miRNAs were markedly enriched (Fig. 4*B*). Notably, miRNA-derived sRNAs constituted approximately 4% of the RISC-bound pool, compared to only 0.3% in total sRNA libraries (Fig. 4*B*), suggesting they are functional. In line with this, the majority of annotated miRNAs harbor RISC sRNAs, strongly suggesting that sRNAs from these loci are loaded into *Ectocarpus* AGO (Fig. 4*C*). Still, a large part of RISC sRNAs originate from intronic and intergenic regions (Fig. 4*B*), suggesting a possible role for these regions as reservoirs of yet unidentified miRNAs or novel classes of regulatory sRNAs in *Ectocarpus*.

To further investigate the impact of the *AGO* mutation on total sRNA accumulation, we identified differentially expressed sRNA loci (DESLs) between the mutant line *ago-1; apt* and the control lines *AGO; apt* and *ago-3; apt* (Fig. 4*D* and Dataset S10). Overall, only a small fraction of sRNA loci were differentially expressed (1.16%; 911 out of 78512), consistent with a role of AGO downstream of sRNA biogenesis. Among these 911 DESLs, only 8 overlapped with DEGs in the *ago* mutants (Dataset S17), representing approximately 2% of all DEGs. This suggests that changes in sRNA production within gene bodies do not generally correlate with the expression levels of the corresponding genes.

In contrast, a significantly higher proportion of annotated miRNAs showed differential expression: 61.0% of Tarver miRNAs (36 out of 59) and 34.2% of putative miRNAs (101 out of 295) (χ^2^ test, *P* < 2.2 × 10^−16^ for both comparisons). Interestingly, downregulated DESLs overlapping with miRNA genes in the *ago-1; apt* mutant compared to the controls were more common than upregulated miRNA DESLs, suggesting that AGO activity may play a role in maintaining or stabilizing miRNA abundance in *Ectocarpus*.

### miRNA Targeting Has a Limited Impact On Transcript Abundance.

**Fig. 5.**
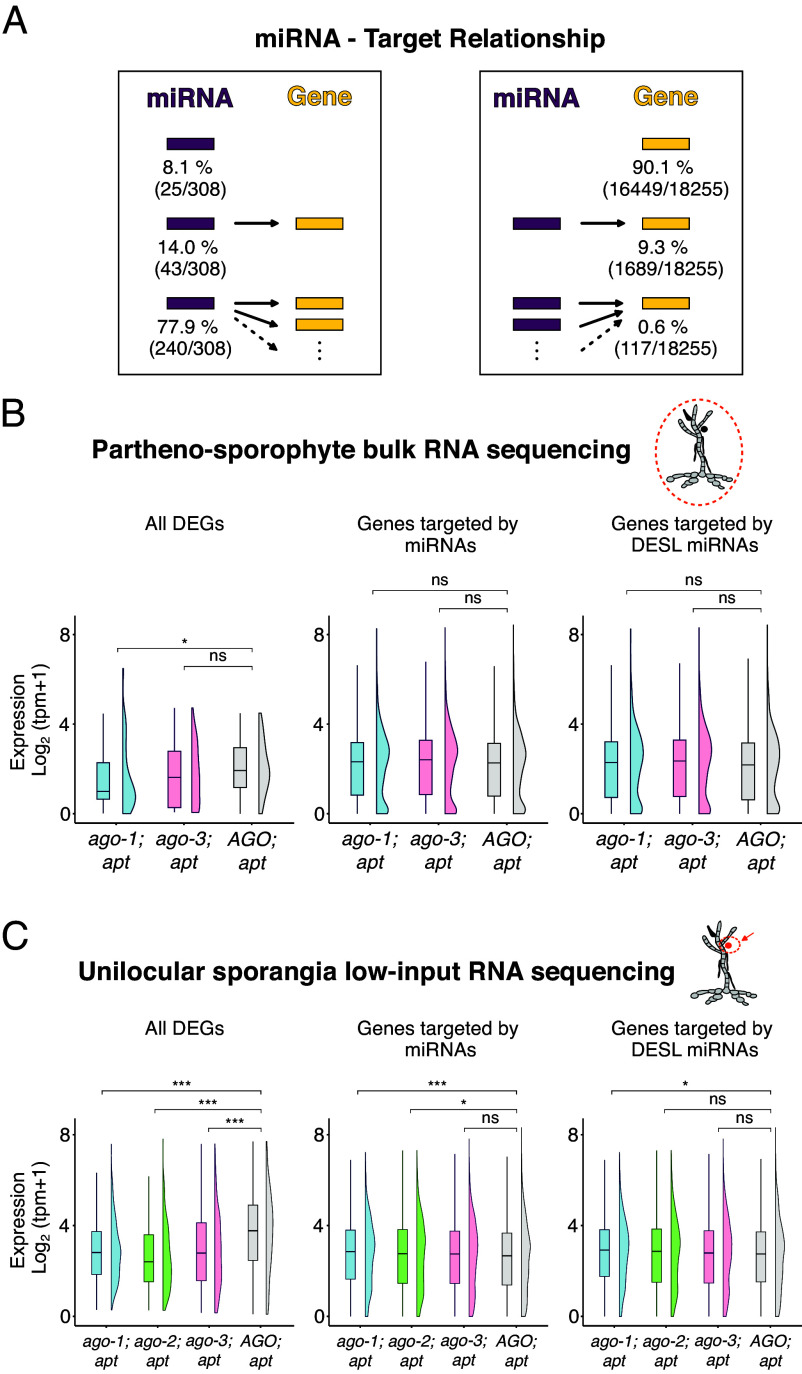
miRNA-target interactions and target gene expression. (*A*) Diagram summarizing the relationships between miRNAs and their predicted target genes. The *Left* panel shows the number of miRNAs that target no genes, one gene, or more than one gene. The *Right* panel shows the number of genes that are not targeted, targeted by one miRNA, or targeted by multiple miRNAs. Target predictions include both Tarver miRNAs and putative miRNAs. (*B*) Gene expression values in bulk mRNA sequencing of partheno-sporophytes from *ago* mutants and the *AGO; apt* control. *Left* panel: Expression levels of DEGs identified in Fig. 3*A*. *Middle* panel: Expression levels of genes predicted to be targeted by Tarver or putative miRNAs. *Right* panel: Expression levels of genes targeted by miRNAs on which a differentially expressed sRNA locus (DESL) was identified (Fig. 4*D*). (*C*) Gene expression values in low-input mRNA sequencing of unilocular sporangia from *ago* mutants and the *AGO; apt* control. *Left* panel: Expression levels of DEGs identified in Fig. 3*C*. *Middle* panel: Expression levels of genes predicted to be targeted by Tarver or putative miRNAs. *Right* panel: Expression levels of genes targeted by miRNAs on which a DESL was identified (Fig. 4*D*). ns = not significant; *P-value < 0.05; ***P-value < 0.001; Wilcoxon test.

miRNAs are known to mediate posttranscriptional gene silencing by guiding AGO proteins to target mRNAs through sequence complementarity, resulting in mRNA cleavage, degradation, or translational repression. To explore the potential regulatory impact of miRNAs in *Ectocarpus*, we determined the targeting spectrum of both Tarver and putative miRNAs by aligning their sequences to the coding sequences of *Ectocarpus* genes, allowing for three mismatches during this process (*Materials and Methods*). Using this approach, we found that 91.9% of miRNAs had at least one identifiable target gene, with 77.9% of these miRNAs targeting two or more genes, and 46.7% targeting five or more genes (Fig. 5*A* and *SI Appendix*, Fig. S5 and Dataset S11 B and C). Conversely, 9.9% of genes in the *Ectocarpus* genome were predicted to be targeted by at least one miRNA, with the vast majority of these genes (9.3%) being targeted by a single miRNA (Fig. 5*A* and Dataset S11A). Overall, these data show that most targeted genes are regulated by a single miRNA, while individual miRNAs tend to have multiple targets, potentially genes with high sequencing homology or belonging to the same functional category or gene family.

Our previous analyses of differential gene expression revealed only limited changes in transcript abundance between *ago* mutants and control lines, suggesting that AGO activity has a modest impact on mRNA abundance (Fig. 3). To assess whether miRNA-target interactions could explain these changes, we examined the relationship between miRNA abundance and the expression of their predicted targets (Dataset S15). Among 38 miRNA-targeted genes that were differentially expressed when comparing nonfunctional mutants (*ago1; apt* or *ago2; apt*) to functional *ago3; apt* mutants or wild-type (*AGO; apt*), only two were consistently deregulated across all low-input datasets, both being downregulated. However, this was not accompanied by changes in the abundance of their targeting miRNAs. One additional gene, encoding a predicted disordered transmembrane protein of unknown function, was upregulated in the bulk dataset concomitant with a decrease in its targeting miRNA, a pattern consistent with miRNA-mediated cleavage (Dataset S15). Nonetheless, this deregulation was not reproducible in the low-input dataset, and while we cannot completely exclude the possibility that this gene contributes to the *ago* phenotype via AGO-mediated transcript cleavage, the lack of consistent deregulation across datasets makes this unlikely.

To further explore the miRNA-target expression relationship in a broader manner, we assess whether genes predicted to be targeted by differentially expressed miRNAs (DESL miRNAs) also show changes in expression, independently of their DEG status. Most DESL miRNAs are downregulated in the *ago-1; apt* mutant compared to the *AGO; apt* control (Fig. 4*D*). If these miRNAs contribute to transcript degradation, we would expect their target genes to be upregulated in the *ago* mutants due to reduced miRNA-mediated silencing. This relationship was not observed when examining gene expression data from both bulk RNA-seq of partheno-sporophytes and low input RNA-seq of unilocular sporangia (Fig. 5 *B* and *C*, panel “Genes targeted by DESL miRNAs”). While a statistically significant difference in expression was observed between *ago-1; apt* and *AGO; apt* in the low-input dataset (with the former showing a slightly higher median expression), this effect was subtle (Fig. 5*B*, panel “Genes targeted by DESL miRNAs”). Moreover, closer inspection of the individual genes contributing to this trend did not reveal consistent upregulation across replicates and between *ago-1; apt* and *ago-2; apt* mutants (Datasets S12 and S13). Therefore, we conclude that there is no robust and consistent evidence that changes in miRNA abundance have a detectable effect on the expression of their predicted target genes under the tested conditions. This finding reinforces our earlier conclusions and strengthens the hypothesis that *Ectocarpus* AGO and its associated miRNAs do not regulate target gene activity through changes in transcript abundance, suggesting instead that regulation occurs via other mechanisms, such as translational repression.

## Discussion

### AGO-Mediated miRNA Function in Ectocarpus.

In this study, we present a comprehensive survey of AGO proteins in brown algae, a diverse and complex group of multicellular organisms in which RNA-based silencing mechanisms remain poorly understood. The phenotypic and molecular descriptions of an *ago* mutant in brown algae have enabled us to investigate the molecular basis of AGO function and to further characterize the sRNA repertoire in this lineage. Consistent with previous findings by Tarver et al. (23), we confirm that the majority of sRNAs in *Ectocarpus* are 20–22 nucleotides in length, with a predominance of 21-nt sRNAs. Extending this analysis, we isolated, RISC-associated sRNAs using the TraPR system (52). The size profile of these AGO-loaded sRNAs closely matched that of the total sRNA population, with a strong enrichment for 21-nt sRNAs bearing a 5′ uridine. This suggests a loading preference of *Ectocarpus* AGO for this sRNA class, resembling the behavior of plant AGO1-clade proteins (53), with which *Ectocarpu*s AGO shares high sequence similarity.

We also observed an enrichment of miRNAs in the RISC-associated sRNA fraction, indicating that these molecules are indeed loaded into the RISC via AGO and suggesting they are functional bona fide miRNAs. A previous survey of sRNA pathway components in *Ectocarpus*, which we have expanded in this study (Dataset S16), identified homologues of several key proteins involved in miRNA function in both plants and animals, suggesting that miRNA biogenesis is possible and broadly similar to that of other eukaryotes (23). These include the *Arabidopsis* proteins TOUGH and SERRATE, which are required for the processing of primary and precursor miRNAs, HASTY, which mediates nuclear export, as well as decapping proteins like AMP1, implicated in mRNA cleavage and translational inhibition (54–57). In contrast, both DROSHA and Pasha are absent from the *Ectocarpus* genome, supporting the idea that miRNA biogenesis in brown algae likely follows a plant-like rather than an animal-like model.

For most sRNA-related proteins identified in *Ectocarpus*, we also detected orthologues in other brown algal species, indicating that these sRNA processing pathways are conserved across the group (Dataset S16). miRNA genes themselves are present in multiple brown algal species, although they show low sequence conservation, suggesting rapid evolutionary turnover within this lineage (58) (Dataset S16). In contrast, diatoms appear to lack canonical miRNAs despite being a closely related Stramenopile lineage, and instead predominantly accumulate sRNAs derived from transposons and repetitive elements (59), suggesting miRNA-mediated gene regulation may have been either lost or never evolved in some Stramenopile lineages.

Our miRNA target prediction analysis estimates that approximately 10% of all *Ectocarpus* genes may be regulated by one or more miRNAs. Nevertheless, despite this broad regulatory potential and the presence of pronounced developmental phenotypes in *ago* mutants, transcriptomic analyses revealed minimal changes in target gene transcript abundance. This suggests that AGO-miRNA-mediated regulation in *Ectocarpus* does not primarily occur through mRNA cleavage, as is common for plant miRNAs, but rather through other mechanisms such as translational repression, better explored in animal systems (13, 14), but also described in plants (56, 60, 61). Together, these findings support a model in which 21-nt miRNAs associate with AGO and are incorporated into the RISC to regulate targets through translational repression. While the precise mechanisms remain to be elucidated, possibilities include inhibition of translation initiation, interference with ribosome progression or recruitment, or sequestration of target mRNAs.

We further observed that the *ago* mutation influences the abundance of mature miRNAs, since in the mutants many miRNAs accumulate to a lower level than the wild-type. This suggests a role for AGO in stabilizing miRNAs after processing or in feedback regulation of their biogenesis. Similar patterns have been observed in plant *ago1* null mutants and to less extent in *ago1* hypomorphic alleles with intact PAZ domains which allows loading and presumably might contribute to miRNA stabilization (62–65) suggesting a feedback mechanism where AGO loading can protect miRNAs from degradation. In animals Ago2 loss-of-function also results in lower mature miRNA abundance (66), further suggesting a conserved role of AGO proteins in miRNA stability or an unknown role in the regulation of miRNA processing efficiency.

### Alternative AGO-Mediated Pathways in *Ectocarpus*.

Among the detected RISC-associated sRNAs, the majority originate from regions not annotated as miRNAs, such as TEs, exons, introns, and intergenic regions. Previous studies have shown that many *Ectocarpus* miRNAs are located within intronic or intergenic regions (23), similar to what is observed in mammals (67, 68). It is therefore possible that a subset of these AGO-loaded intronic/intergenic sRNAs represent bona fide miRNAs that remain unidentified due to current annotation limitations. Alternatively, this enrichment may reflect the genomic context of other, yet uncharacterized, classes of regulatory sRNAs.

Although our analyses focused primarily on miRNA-mediated AGO function, we cannot exclude the possibility that other sRNA classes underlie the *ago* mutant phenotype. Such mechanisms could involve siRNA-mediated TGS through chromatin modification, or PTGS via siRNA-directed transcript cleavage, rather than canonical miRNA activity. Another possibility is that AGO acts transcriptionally, through sRNA-guided association with chromatin in cooperation with the SWI/SNF complex, as reported for *Arabidopsis* AGO1 (51), where siRNA accumulation at target genes promotes AGO1 recruitment and transcriptional activation.

However, all these models would be expected to produce detectable and specific transcriptional changes, which we do not observe in our dataset. Therefore, our results instead suggest that the *Ectocarpus* AGO acts primarily through translational regulation, potentially mediated by miRNAs or other RISC-associated sRNAs.

### A Conserved Role for AGO in Life Cycle Progression and Germline Competence.

We show that *Ectocarpus* AGO mutants exhibit a severe defect in the production of viable meiospores, which are essential for establishing the gametophyte generation, ultimately responsible for producing gametes. This phenotype results in a drastic reduction in sexual reproductive fitness: in *ago* mutants, most meiospores are inviable and fail to germinate, and in the cases where germination occurs, the resulting individuals develop as functional partheno-sporophytes and fail the transition into the reproductive gametophyte phase, effectively blocking germline establishment and gamete production. Microscopy observations showed that meiospore morphology remains unchanged in the mutants, suggesting that meiosis proceeds normally and that the developmental arrest occurs downstream of meiotic division. These observations point to a failure in the expression or function of proteins required for meiospore viability, germination, and the establishment of gametophytic/germline cell identity, potentially due to the loss of AGO-mediated translational regulation. This is consistent with roles described for AGO proteins in other organisms, where they affect the specification and maintenance of reproductive cell fate. For example, in *Drosophila*, AGO1 is required in germline stem cells to prevent premature differentiation (69). In plants, several AGO proteins regulate meiosis and gamete formation, as demonstrated by MEL1 in rice (70) and AGO104 in maize (71). Similarly, in the fungus *Sordaria macrospora*, AGO proteins are required for meiocyte formation and progression into the sexual cycle (72).

Interestingly, we observed that the meiospore defect in absence of AGO is determined not by the genotype of the meiospore itself but by that of the sporophyte in which the spores are formed. This suggests a non-cell-autonomous function of AGO, possibly involving interactions with sRNAs in the surrounding sporophytic tissue that influence meiospore viability and identity. Similar non-cell-autonomous roles for AGO proteins have been described in both plants and animals, where accessory or somatic cells produce sRNAs that, through AGO proteins, safeguard genome integrity and regulate TE activity in adjacent reproductive cells. In *Arabidopsis*, for instance, AGO9 is active in somatic companion cells of the ovule where it interacts with 24-nt siRNAs derived from TEs, and its activity is required to ensure proper female gamete fate specification (40). In pollen, AGO-dependent small RNAs are produced in companion cells and transported to the sperm cells to reinforce TE silencing (73). In animals, PIWI-clade proteins in somatic support cells, such as Piwi in *Drosophila* follicle cells (74) and MIWI2/MILI in mammalian Sertoli cells (75) mediate piRNA production and TE silencing in the germline.

Our results could reflect an AGO-dependent activity established in diploid sporophytic cells and inherited through meiosis. This activity could correspond to RISCs or to downstream regulatory outputs such as stabilized or repressed mRNAs established before meiosis. Such inheritance would allow all *ago* meiospores derived from a heterozygous diploid sporophyte to develop normally, consistent with our observation of complete rescue. Similarly, in *Drosophila* maternally inherited previously assembled Piwi–piRNA complexes initiate transposon silencing in the embryo (76).

Together, these findings highlight a fundamental role for a brown algal AGO in regulating the key developmental transition from sporophyte to gametophyte, ensuring competence for sexual reproduction, and support a broadly conserved function for AGO proteins in germline specification and reproductive development across eukaryotes.

### A Streamlined AGO and sRNA Repertoire in Brown Algae.

A striking finding from our study is that all surveyed brown algae possess only a single AGO gene in their genomes. This contrasts with most complex multicellular eukaryotes, which typically encode multiple AGO paralogs with specialized and often nonredundant roles in different RNA silencing pathways (1–3). The presence of just one AGO in brown algae raises interesting questions about the functional versatility of this protein and the overall architecture of RNA silencing in this lineage.

One possibility is that the brown algal AGO is particularly versatile and capable of mediating a wide range of RNA silencing processes. However, the observed sRNA landscape in *Ectocarpus*, particularly the strong enrichment for 21-nt sRNAs, especially those loaded into RISCs, suggests a relatively streamlined silencing system. In many other systems, specific AGOs preferentially bind distinct sRNA species: For example, AGO1 in *Arabidopsis* primarily loads 21-nt miRNAs, AGO4 associates with 24-nt siRNAs involved in transcriptional repression and genome integrity, and PIWI proteins in animals interact with 21 to 31-nt piRNAs crucial for transposon repression in the germline (16, 18). In our study, we observed that approximately 15% of RISC-associated sRNAs in *Ectocarpus* originate from TEs, potentially indicating a role for AGO in regulating TE activity. This is in line with a recent study that reported both sRNAs and the histone mark H3K79me2 are associated with active TEs in *Ectocarpus* (77), suggesting a potential relationship between sRNA activity and histone mark deposition. Interestingly, TE-derived sRNAs fall within the 21-nt size class, suggesting that if AGO-mediated TE silencing occurs in *Ectocarpus*, it likely operates through this core sRNA class rather than a specialized TE-targeting sRNA population. The lack of both AGO diversification and sRNA class specialization highlights a simplified, but potentially highly adapted RNA silencing system in brown algae.

Despite this apparent reduction in complexity, the *Ectocarpus* AGO retains the canonical PAZ, MID, and PIWI domains, including a conserved DEDH catalytic tetrad with capacity for slicing/endonuclease activity. Interestingly, all examined brown algal AGOs also exhibit a long, intrinsically disordered N-terminal extension. In other systems, such disordered regions have been shown to influence subcellular localization, mediate interactions with regulatory proteins, and serve as targets for posttranslational modifications (78–81). It is conceivable that in brown algae, the N-terminal region contributes to the regulation and versatility of the single AGO protein, enabling it to participate in diverse functions, such as developmental transitions, as highlighted in this study, and potentially in stress or defense responses—functions that in other lineages require multiple AGO paralogs.

## Materials and Methods

### Biological Material.

The strains used in this study are described in Dataset S2. The male *Ectocarpus* species 7 strain Ec32 was used as a background for all CRISPR/Cas experiments, for which a reference genome is available (82). Although strain Ec32 was previously referred to as *E. siliculosus,* it belongs to a distinct, at present unnamed, species (83), which is referred to provisionally as *Ectocarpus* species 7 (*Ectocarpus* sp.7). For simplicity, we use the term *Ectocarpus* here. Strains were cultured at 14 °C with a light: dark cycle of 12 h (30 mmol m^−2^ s^−1^) and daylight-type LEDs (adapted from refs. 48 and 84). All manipulations were performed under sterile conditions in a laminar flow hood.

### CRISPR/Cas Mutagenesis.

We followed the protocols described previously (47, 48), using guides to target *AGO (Ec-01_00900)* and *APT (Ec-28_000520)* in *Ectocarpus*. *APT* codes for an adenine phosphoribosyl transferase and is used as a selective marker, since loss-of-function *apt* mutants lead to a resistant phenotype when algae are grown in a medium containing the toxic compound 2-fluoroadenine. As a result, all *ago* mutants identified also carry a mutation at the *APT* locus (Dataset S2). It is important to note that *apt* mutant lines exhibit significant changes in their global transcriptomes (48); thus, all comparisons with *ago* mutants were made using *AGO; apt* lines as controls. Mutations were identified via PCR amplification followed by Sanger sequencing. Guide RNAs and PCR primers are listed in Dataset S3.

### Microscopy.

Bright-field imaging of all life stages in *ago* mutants and their respective controls was performed in a Zeiss Axiovert microscope.

Fluorescent imaging of unilocular sporangia was performed on a Zeiss Observer microscope. Samples were stained with DAPI, mounted on a glass slide, and subsequently imaged using a chlorophyll filter to detect chloroplast autofluorescence, a FITC filter to detect flavin autofluorescence on the posterior flagella, and a DAPI filter. DIC images were also obtained for these samples.

Transmission electron microscopy (TEM) was used to further characterize the cellular architecture of *ago* mutant and control meiospores. Filaments with unilocular sporangia were high-pressure frozen (HPF Compact 03, Engineering Office M. Wohlwend GmbH), freeze-substituted (AFS2, Leica Microsystems) with 0.2% osmium tetroxide and 0.1% uranyl acetate in acetone containing 1.5% water as substitution medium, and embedded in Epon. Sections were stained with uranyl acetate and lead citrate and analyzed with a Tecnai Spirit (Thermo Fisher Scientific) operated at 120 kV or a JEM-2100 Plus (Jeol Germany GmbH) operated at 200 kV.

### Phenotyping of Unilocular Sporangia.

To assess the viability of unilocular sporangia and meiospore fate in *ago* mutant and *AGO; apt* control strains, individual unilocular sporangia were microdissected from mature partheno-sporophytes. The viability of unilocular sporangia was assessed 24 to 48 h after dissection when unilocular sporangia of control algae normally release their meiospore content. Individual released meiospores of each strain were then assessed for viability and further phenotyped when clear morphological differentiation between gametophytes and partheno-sporophytes was evident: 7th and 10th day postrelease for control strains and 2.5 to 4 wk for the mutants, as these latter developed slightly slower.

### Crosses.

Genetic crosses were performed as described previously (27, 85). Although most ago mutant meiospores were inviable or wrongly developed into partheno-sporophytes in *ago* mutants, we exploited an escapee *ago-1; apt* gametophyte and used it to perform genetic crosses (*SI Appendix*, Fig. S4 and Dataset S5). The *ago-1; apt* mutant gametophyte was crossed with the wild-type female line Ec25 to generate a diploid sporophyte. From this sporophyte, a segregating population of 38 gametophyte individuals was produced, each derived from a different unilocular sporangium (Dataset S5). This segregating population was genotyped (primers are specified in Dataset S3) and phenotyped using an inverted microscope.

### mRNA Sequencing.

For bulk RNA-seq, RNA was extracted from triplicate samples, each containing at least 800 mature *ago-1; apt*, *ago-3; apt* or *AGO; apt* partheno-sporophytes. Algae were snap-frozen in liquid nitrogen and stored at –80 °C prior to RNA extraction. Samples were dry-ground in precooled mortars using liquid nitrogen until a fine gray-white powder was obtained. 1 mL of CTAB3 extraction buffer was prepared as follows: 350 µL RNase-free H_2_O, 100 µL Tris-HCl (pH 8.0, to a final concentration of 100 mM), 280 µL NaCl (final 1.4 M), 40 µL EDTA (pH 8.0, final 20 mM), 20 µL Plant RNA Isolation Aid (PVP) (2%), 200 µL CTAB (2% final), and 10 µL β-mercaptoethanol (1% final). 700 to 750 μL of preheated CTAB3 buffer (65 °C) was added to the powdered tissue, vortexed briefly, spun down, and incubated at 65 °C for 1 to 2 min. An equal volume of chloroform:isoamyl alcohol (24:1) was added, mixed vigorously, and centrifuged at 10,000×*g* for 15 min at 4 °C. The upper aqueous phase was transferred to a new RNase-free tube. The chloroform extraction was repeated once. The final aqueous phase was transferred to a 1.5 mL RNase-free tube, and RNA was precipitated by adding an equal volume of isopropanol. Samples were mixed thoroughly and incubated at –20 °C overnight (up to 72 h). RNA was pelleted by centrifugation at maximum speed for 45 to 60 min at 4 °C, followed by washing with 1 mL of freshly prepared, ice-cold 70% ethanol. Pellets were air-dried briefly (3 to 5 min) and then dissolved in 25 to 50 μL of RNase-free water. DNase treatment was performed using the TURBO DNase Kit (Thermo Fisher, AM1907). RNA concentration was assessed using a Nanodrop spectrophotometer (acceptable: A260/A280 ≥ 2.0, A260/A230 ≥ 2.0, RNA concentration ≤ 200 ng/μL). RNA was further purified using the Zymo RNA Clean & Concentrator Kit (Zymo Research, R1013) with additional washing steps: two washes with RNA-Prep buffer (400 μL) and four washes with RNA wash buffer (700 μL). as described previously (86, 87). For each replicate, the RNA was quantified and cDNA was synthesized using an oligo-dT primer. The cDNA was fragmented, cloned, and sequenced in an Illumina NextSeq 2000 platform.

Low input RNA-seq was performed on triplicate microdissected unilocular sporangia samples of mature *ago-1; apt*, *ago-2; apt, ago-3; apt* and *AGO; apt* partheno-sporophytes. The material was flash frozen, and RNA extraction, library preparation, and sequencing were done as described previously (48, 88).

### Total sRNA Sequencing.

Total sRNA sequencing was performed in the exact same samples as the bulk mRNA sequencing—described above. Library preparation and sequencing were carried out by Novogene. In brief, the library construction included the ligation of 5′ and 3′ adaptors to the sRNA ends and a first-strand cDNA synthesis after hybridization with a reverse transcription primer. Double-stranded cDNA libraries were enriched by PCR. Fragments containing inserts between ∼18 and 40 bp were size selected and purified prior to Illumina sequencing to generate 50-bp single-end reads. Samples were sequenced with single-end 50 bp reads on an Illumina NextSeq platform.

### RISC sRNA Sequencing.

RISC-associated sRNAs were isolated using the TraPR sRNA isolation kit form Lexogen (Cat. No. 128) with minor modifications: 25 mg mature *AGO; apt* patheno-sporophyte were collected and snap-frozen and then ground into a fine powder using a liquid nitrogen precooled pestle in a low-bind 1,5 mL Eppendorf tube. 750 µL TLB buffer was added into the tube to homogenize the sample. The lysate was clarified by centrifugation at 4 °C with 10,000×*g* for 5 min. Each 250 µL clarified lysate was loaded onto one prepared TraPR column and mixed well. Three columns were used for a total 750 µL clarified lysate. During elution, bulk RNA and DNA were retained in the columns and the RISC fraction containing AGO-bound sRNAs was eluted 3× with 250 µL of TEB buffer for a total eluate volume of 750 µL per column. Eluates from the three columns were pooled for phenol/chloroform/isoamylalcohol extraction to isolate sRNAs, which were then used for library preparation following the Small RNA-Seq Library Prep Kit for Illumina with TraPR (Lexogen, catalog number 135) with slight changes: During the library construction, the amount of 3′ adapter, 5′ adapter, and reverse transcription primer was decreased to 0, 1×, and library amplification was done for 20 PCR cycles. Samples were sequenced with single-end 50 bp reads on an Illumina NextSeq platform.

### mRNA Mapping and DEGs.

Raw reads were processed using Trimmomatic (89) for quality control and adapter removal. A threshold of 3 was applied to trim low-quality bases from both the beginning and end of each read. To address potential sequencing artifacts, the first 10 bases of each read were removed. Additionally, reads shorter than 50 bases were discarded to maintain overall data quality.

PolyA trimming was performed using PRINSEQ (90). During this step, up to 5 consecutive A or T bases were removed from the 5′ and 3′ ends of the reads. Reads with a minimum length of 30 bases were retained for further analysis. Single-end and paired-end reads were processed separately during this step.

High-quality reads from each library were subsequently mapped to the *Ectocarpus* Ec32 transcriptome reference (82) using the STAR aligner (91). Two independent sequencing runs were conducted, and reads from the corresponding libraries were combined in this step for downstream analyses.

Gene expression quantification was performed using featureCounts (92), which counted the number of reads mapped to each gene. After this step, the counts from single-end and paired-end reads were summed for the corresponding samples, resulting in a raw count matrix.

Pairwise differential expression between the mutants was obtained using raw read count matrices as input for the DESeq2 package (93) in R v.4.3.1. *P*-values were adjusted for multiple testing using the Benjamini–Hochberg method to control the false discovery rate. DEGs were identified based on an adjusted *P*-value threshold of 0.001. Additionally, only genes exhibiting a minimum log fold change of 1 between mutants were considered significant.

### sRNA Mapping and Analysis.

Total and RISC sRNA reads were mapped using Bowtie v1.2.3 (94) to the *Ectocarpus* Ec32 male genome (82), as described previously (31). The following parameters were set: --wrapper basic-0 -v 0 -S. No mismatches were allowed. The sRNA read length was extracted from the BAM file. According to the sRNA read length, the first nucleotide of each read was counted and presented in a histogram. The genomic origin of total and RISC sRNA was assigned according to the gene model annotation of Liu et al. (82), the TE annotation of Dinatale et al. (77), and the miRNA gene annotation described below.

### miRNA Gene Annotation.

Two sets of miRNA gene annotations were used in this study: Tarver miRNAs [corresponding to those miRNAs identified previously (23)], as well as putative miRNAs. To create the set of putative miRNAs we used a combination of the miRNA finding methods described previously (23) together with a palindrome-based finding strategy: All > 8 bp palindromic regions at the *Ectocarpus* genome were collected and the middle position of the palindrome was extended by 70 bp to both directions. These regions were then intersected with positions of mapped sRNA reads, and only palindromes containing mapped sRNAs with a coverage higher than 20× were kept. These potential miRNA gene regions were then manually curated by visual inspection on JBrowse (95). The putative miRNA gene was kept if the typical miRNA pattern of a unique miRNA and miRNA* was present. Duplicated putative miRNAs at different locations on the genome were annotated as individual miRNA gene models. Application of these criteria led to the retention of 295 putative miRNAs (Dataset S9).

### DESL.

To identify DESL, sRNA abundance was measured in different genomic features: Genes present on the gene model annotation of Liu et al. (82), including miRNA genes defined by Tarver et al. (23) and putative miRNAs identified in this study, as well as intergenic regions. To obtain the latter, unannotated intergenic regions were divided into 5 kb bins and each of these features was named using a combination of the upstream gene ID and a unique number. This approach was done for the forward and the reverse strand. featureCounts v2.0.3 (options: -s 1 -d 15 -M -a) (92) was then used to generate a sRNA read count matrix. Differential expression analysis was then performed by the R (4.4.1) package DESeq2 v1.44.0 (93), and all features with a statistically significant differential sRNA abundance were categorized as DESLs (96).

### Phylogenetic Analysis of AGO Proteins.

To identify divergent homologs of the *Ectocarpus* AGO protein, we queried the profile hidden Markov model (HMM) database of the *Ectocarpus* sp. 7 proteome using HHsearch with default parameters (43). In addition, BLAST (97) searches at NCBI were performed to identify eukaryotic AGO sequences with the highest similarity to the brown algal AGOs. Structural predictions for representative eukaryotic AGOs were generated with AlphaFold3 (98). Where experimentally determined structures were available, predicted models were validated by structural superposition, typically yielding RMSDs of less than 1 Å, indicating excellent predictive accuracy.

For phylogenetic analysis, we employed the PhyML+SMS/OneClick workflow available at the NGPhylogeny.fr web server (99, 100). Eukaryotic AGO and PIWI-like representatives were selected based on the set characterized in (101) and supplemented with brown algal AGO sequences from *Ectocarpus siliculosus*, *C. okamuranus*, *F. serratus*, *D. dichotoma*, and *N. decipiens*, as well as from the closely related yellow-green alga *T. minus*. The final dataset comprised 199 sequences (full-length protein sequences are provided in the Dataset S1). Prior to phylogenetic inference, the N-terminal intrinsically disordered region was removed from each sequence based on AlphaFold3 predictions. Sequence alignment (MAFFT L-INS-i), trimming (BMGE), model selection, and tree inference (SPR; SH-like aLRT support) were performed within the OneClick workflow. The resulting tree was visualized in iTOL (102).

## Supplementary Material

Appendix 01 (PDF)

Dataset S01 (XLSX)

Dataset S02 (XLSX)

Dataset S03 (XLSX)

Dataset S04 (XLSX)

Dataset S05 (XLSX)

Dataset S06 (XLSX)

Dataset S07 (XLSX)

Dataset S08 (XLSX)

Dataset S09 (XLSX)

Dataset S10 (XLSX)

Dataset S11 (XLSX)

Dataset S12 (XLSX)

Dataset S13 (XLSX)

Dataset S14 (XLSX)

Dataset S15 (XLSX)

Dataset S16 (XLSX)

Dataset S17 (XLSX)

## Data Availability

All the sequencing data obtained in this study are summarized in Dataset S14 and deposited in the NCBI SRA BioProject PRJNA1294542 (103). All other data are included in the manuscript and/or supporting information.

## References

[1] J. Höck, G. Meister, The argonaute protein family. Genome Biol. **9**, 210 (2008). 18304383 10.1186/gb-2008-9-2-210 PMC2374724

[2] G. Hutvagner, M. J. Simard, Argonaute proteins: Key players in RNA silencing. Nat. Rev. Mol. Cell Biol. **9**, 22–32 (2008). 18073770 10.1038/nrm2321

[3] G. Meister, Argonaute proteins: Functional insights and emerging roles. Nat. Rev. Genet. **14**, 447–459 (2013). 23732335 10.1038/nrg3462

[4] P. Bobadilla Ugarte, P. Barendse, D. C. Swarts, Argonaute proteins confer immunity in all domains of life. Curr. Opin. Microbiol. **74**, 102313 (2023). 37023508 10.1016/j.mib.2023.102313

[5] A. Mallory, H. Vaucheret, Form, function, and regulation of ARGONAUTE proteins. Plant Cell **22**, 3879–3889 (2010). 21183704 10.1105/tpc.110.080671 PMC3027166

[6] B. Koopal, S. K. Mutte, D. C. Swarts, A long look at short prokaryotic Argonautes. Trends Cell Biol. **33**, 605–618 (2023). 36428175 10.1016/j.tcb.2022.10.005

[7] D. C. Swarts , The evolutionary journey of Argonaute proteins. Nat. Struct. Mol. Biol. **21**, 743–753 (2014). 25192263 10.1038/nsmb.2879 PMC4691850

[8] L. Peters, G. Meister, Argonaute proteins: Mediators of RNA silencing. Mol. Cell **26**, 611–623 (2007). 17560368 10.1016/j.molcel.2007.05.001

[9] H. Zhang, R. Xia, B. C. Meyers, V. Walbot, Evolution, functions, and mysteries of plant ARGONAUTE proteins. Curr. Opin. Plant Biol. **27**, 84–90 (2015). 26190741 10.1016/j.pbi.2015.06.011

[10] J. Liu , Argonaute2 is the catalytic engine of mammalian RNAi. Science **305**, 1437–1441 (2004). 15284456 10.1126/science.1102513

[11] K. Bohmert , *AGO1* defines a novel locus of *Arabidopsis* controlling leaf development. EMBO J. **17**, 170–180 (1998). 9427751 10.1093/emboj/17.1.170 PMC1170368

[12] J.-B. Morel , Fertile hypomorphic ARGONAUTE (ago1) mutants impaired in post-transcriptional gene silencing and virus resistance. Plant Cell **14**, 629–639 (2002). 11910010 10.1105/tpc.010358 PMC150585

[13] D. Baulcombe, RNA silencing in plants. Nature **431**, 356–363 (2004). 15372043 10.1038/nature02874

[14] M. Ghildiyal, P. D. Zamore, Small silencing RNAs: An expanding universe. Nat. Rev. Genet. **10**, 94–108 (2009). 19148191 10.1038/nrg2504 PMC2724769

[15] V. N. Kim, J. Han, M. C. Siomi, Biogenesis of small RNAs in animals. Nat. Rev. Mol. Cell Biol. **10**, 126–139 (2009). 19165215 10.1038/nrm2632

[16] D. M. Ozata, I. Gainetdinov, A. Zoch, D. O’Carroll, P. D. Zamore, PIWI-interacting RNAs: Small RNAs with big functions. Nat. Rev. Genet. **20**, 89–108 (2019). 30446728 10.1038/s41576-018-0073-3

[17] C. Juliano, J. Wang, H. Lin, Uniting germline and stem cells: The function of Piwi proteins and the piRNA pathway in diverse organisms. Annu. Rev. Genet. **45**, 447–469 (2011). 21942366 10.1146/annurev-genet-110410-132541 PMC3832951

[18] X. Fang, Y. Qi, RNAi in plants: An argonaute-centered view. Plant Cell **28**, 272–285 (2016). 26869699 10.1105/tpc.15.00920 PMC4790879

[19] N. Baumberger, D. C. Baulcombe, *Arabidopsis* ARGONAUTE1 is an RNA Slicer that selectively recruits microRNAs and short interfering RNAs. Proc. Natl. Acad. Sci. U.S.A. **102**, 11928–11933 (2005). 16081530 10.1073/pnas.0505461102 PMC1182554

[20] D. Cuerda-Gil, R. K. Slotkin, Non-canonical RNA-directed DNA methylation. Nat. Plants **2**, 1–8 (2016). 10.1038/nplants.2016.163 27808230

[21] M. A. Matzke, R. A. Mosher, RNA-directed DNA methylation: An epigenetic pathway of increasing complexity. Nat. Rev. Genet. **15**, 394–408 (2014). 24805120 10.1038/nrg3683

[22] F. Denoeud , Evolutionary genomics of the emergence of brown algae as key components of coastal ecosystems. Cell **187**, 6943–6965.e39 (2024). 39571576 10.1016/j.cell.2024.10.049

[23] J. E. Tarver , MicroRNAs and the evolution of complex multicellularity: Identification of a large, diverse complement of microRNAs in the brown alga *Ectocarpus*. Nucleic Acids Res. **43**, 6384–6398 (2015). 26101255 10.1093/nar/gkv578 PMC4513859

[24] J. M. Cock, O. Godfroy, N. Macaisne, A. F. Peters, S. M. Coelho, Evolution and regulation of complex life cycles: A brown algal perspective. Curr. Opin. Plant Biol. **17**, 1–6 (2014). 24507487 10.1016/j.pbi.2013.09.004

[25] S. M. Coelho, The brown seaweed *Ectocarpus*. Nat. Methods **21**, 363–364 (2024). 38472460 10.1038/s41592-024-02198-6

[26] O. Godfroy , DISTAG/TBCCd1 is required for basal cell fate determination in *Ectocarpus*. Plant Cell **29**, 3102–3122 (2017). 29208703 10.1105/tpc.17.00440 PMC5757272

[27] O. Godfroy , The baseless mutant links protein phosphatase 2A with basal cell identity in the brown alga *Ectocarpus*. Development **150**, dev201283 (2023). 36786333 10.1242/dev.201283 PMC10112911

[28] A. Arun , Convergent recruitment of TALE homeodomain life cycle regulators to direct sporophyte development in land plants and brown algae. eLife **8**, e43101 (2019). 30644818 10.7554/eLife.43101 PMC6368402

[29] S. M. Coelho , Ouroboros is a master regulator of the gametophyte to sporophyte life cycle transition in the brown alga Ectocarpus. Proc. Natl. Acad. Sci. U.S.A. **108**, 11518–11523 (2011). 21709217 10.1073/pnas.1102274108 PMC3136289

[30] S. Bourdareau , Histone modifications during the life cycle of the brown alga *Ectocarpus*. Genome Biol. **22**, 12 (2021). 33397407 10.1186/s13059-020-02216-8 PMC7784034

[31] J. Vigneau , Interactions between U and V sex chromosomes during the life cycle of *Ectocarpus*. Development **151**, dev.202677 (2024). 10.1242/dev.202677 PMC11057875 38512707

[32] J. H. Bothwell, D. Marie, A. F. Peters, J. M. Cock, S. M. Coelho, Role of endoreduplication and apomeiosis during parthenogenetic reproduction in the model brown alga *Ectocarpus*. New Phytol. **188**, 111–121 (2010). 20618911 10.1111/j.1469-8137.2010.03357.x

[33] E. I. Kane, L. S. Trefs, L. Eckert, S. M. Coelho, J. R. Weir, Characterization of meiotic axis proteins in the model brown alga *Ectocarpus*. EMBO Rep. **26**, 5673–5702 (2025). 41131160 10.1038/s44319-025-00605-3 PMC12678776

[34] F. Berger, D. Twell, Germline specification and function in plants. Annu. Rev. Plant Biol. **62**, 461–484 (2011). 21332359 10.1146/annurev-arplant-042110-103824

[35] E. E. Saffman, P. Lasko, Germline development in vertebrates and invertebrates. Cell. Mol. Life Sci. **55**, 1141–1163 (1999). 10442094 10.1007/s000180050363 PMC11147032

[36] V. Walbot, On the life strategies of plants and animals. Trends Genet. **1**, 165–169 (1985).

[37] F. Borges, R. A. Martienssen, The expanding world of small RNAs in plants. Nat. Rev. Mol. Cell Biol. **16**, 727–741 (2015). 26530390 10.1038/nrm4085 PMC4948178

[38] S. Feng, S. E. Jacobsen, W. Reik, Epigenetic reprogramming in plant and animal development. Science **330**, 622–627 (2010). 21030646 10.1126/science.1190614 PMC2989926

[39] M. Hemberger, W. Dean, W. Reik, Epigenetic dynamics of stem cells and cell lineage commitment: Digging Waddington’s canal. Nat. Rev. Mol. Cell Biol. **10**, 526–537 (2009). 19603040 10.1038/nrm2727

[40] V. Olmedo-Monfil , Control of female gamete formation by a small RNA pathway in *Arabidopsis*. Nature **464**, 628–632 (2010). 20208518 10.1038/nature08828 PMC4613780

[41] R. K. Slotkin , Epigenetic reprogramming and small RNA silencing of transposable elements in pollen. Cell **136**, 461–472 (2009). 19203581 10.1016/j.cell.2008.12.038 PMC2661848

[42] I. J. MacRae , Structural basis for double-stranded RNA processing by dicer. Science **311**, 195–198 (2006). 16410517 10.1126/science.1121638

[43] L. Zimmermann , A completely reimplemented MPI bioinformatics toolkit with a new HHpred server at its core. J. Mol. Biol. **430**, 2237–2243 (2018). 29258817 10.1016/j.jmb.2017.12.007

[44] A. Wallmann, M. Van de Pette, Structural and evolutionary determinants of argonaute function. Nucleic Acids Res. **53**, gkaf962 (2025). 41020504 10.1093/nar/gkaf962 PMC12477606

[45] H. Nakayashiki, N. Kadotani, S. Mayama, Evolution and diversification of RNA silencing proteins in fungi. J. Mol. Evol. **63**, 127–135 (2006). 16786437 10.1007/s00239-005-0257-2

[46] D. G. Müller, Sex expression in aneuploid gametophytes of the brown alga *Ectocarpus siliculosus* (Dillw.) Lyngb. Arch. Protistenk. **117**, 297–302 (1975).

[47] C. Martinho , Efficient CRISPR Cas genome editing in brown algae. Cell Rep Methods, 10.1016/j.crmeth.2025.101273 (2025). PMC12853178 41475352

[48] R. Luthringer , Repeated co-option of HMG-box genes for sex determination in brown algae and animals. Science **383**, eadk5466 (2024). 38513029 10.1126/science.adk5466

[49] S. M. Coelho, A. F. Peters, D. Müller, J. M. Cock, *Ectocarpus*: An evo-devo model for the brown algae. EvoDevo **11**, 19 (2020). 32874530 10.1186/s13227-020-00164-9 PMC7457493

[50] A. F. Peters , Life-cycle-generation-specific developmental processes are modified in the immediate upright mutant of the brown alga *Ectocarpus siliculosus*. Development **135**, 1503–1512 (2008). 18339673 10.1242/dev.016303

[51] C. Liu , *Arabidopsis* ARGONAUTE 1 binds chromatin to promote gene transcription in response to hormones and stresses. Dev. Cell **44**, 348–361.e7 (2018). 29290588 10.1016/j.devcel.2017.12.002

[52] T. Grentzinger , A universal method for the rapid isolation of all known classes of functional silencing small RNAs. Nucleic Acids Res. **48**, e79 (2020). 32496553 10.1093/nar/gkaa472 PMC7641303

[53] S. Mi , Sorting of small RNAs into Arabidopsis Argonaute complexes is directed by the 5′ terminal nucleotide. Cell **133**, 116–127 (2008). 18342361 10.1016/j.cell.2008.02.034 PMC2981139

[54] M. Jozwiak, D. Bielewicz, Z. Szweykowska-Kulinska, A. Jarmolowski, M. Bajczyk, Serrate: A key factor in coordinated RNA processing in plants. Trends Plant Sci. **28**, 841–853 (2023). 37019716 10.1016/j.tplants.2023.03.009

[55] K. Motomura , The role of decapping proteins in the miRNA accumulation in *Arabidopsis thaliana*. RNA Biol. **9**, 644–652 (2012). 22614834 10.4161/rna.19877

[56] S. Li , MicroRNAs inhibit the translation of target mRNAs on the endoplasmic reticulum in *Arabidopsis*. Cell **153**, 562–574 (2013). 23622241 10.1016/j.cell.2013.04.005 PMC3694718

[57] F. Brioudes , HASTY, the *Arabidopsis* EXPORTIN5 ortholog, regulates cell-to-cell and vascular microRNA movement. EMBO J. **40**, e107455 (2021). 34152631 10.15252/embj.2020107455 PMC8327949

[58] J. M. Cock , Rapid evolution of microRNA loci in the brown algae. Genome Biol. Evol. **9**, 740–749 (2017). 28338896 10.1093/gbe/evx038 PMC5381526

[59] S. Lopez-Gomollon , Global discovery and characterization of small non-coding RNAs in marine microalgae. BMC Genomics **15**, 697 (2014). 25142467 10.1186/1471-2164-15-697 PMC4156623

[60] H. Iwakawa, Y. Tomari, Molecular insights into microRNA-mediated translational repression in plants. Mol. Cell **52**, 591–601 (2013). 24267452 10.1016/j.molcel.2013.10.033

[61] E. Lanet , Biochemical evidence for translational repression by *Arabidopsis* microRNAs. Plant Cell **21**, 1762–1768 (2009). 19531599 10.1105/tpc.108.063412 PMC2714937

[62] C. Speth, E.-M. Willing, S. Rausch, K. Schneeberger, S. Laubinger, *RACK1* scaffold proteins influence miRNA abundance in *Arabidopsis*. Plant J. **76**, 433–445 (2013). 23941160 10.1111/tpj.12308

[63] Y. Kurihara , Transcriptome analyses revealed diverse expression changes in *ago1* and *hyl1* *Arabidopsis* mutants. Plant Cell Physiol. **50**, 1715–1720 (2009). 19633021 10.1093/pcp/pcp109

[64] H. Vaucheret, F. Vazquez, P. Crété, D. P. Bartel, The action of ARGONAUTE1 in the miRNA pathway and its regulation by the miRNA pathway are crucial for plant development. Genes Dev. **18**, 1187–1197 (2004). 15131082 10.1101/gad.1201404 PMC415643

[65] A. Lingel, B. Simon, E. Izaurralde, M. Sattler, Structure and nucleic-acid binding of the *Drosophila* Argonaute 2 PAZ domain. Nature **426**, 465–469 (2003). 14615801 10.1038/nature02123

[66] J. Winter, S. Diederichs, Argonaute proteins regulate microRNA stability: Increased microRNA abundance by argonaute proteins is due to microRNA stabilization. RNA Biol. **8**, 1149–1157 (2011). 21941127 10.4161/rna.8.6.17665

[67] Y.-K. Kim, V. N. Kim, Processing of intronic microRNAs. EMBO J. **26**, 775–783 (2007). 17255951 10.1038/sj.emboj.7601512 PMC1794378

[68] A. Rodriguez, S. Griffiths-Jones, J. L. Ashurst, A. Bradley, Identification of mammalian microRNA host genes and transcription units. Genome Res. **14**, 1902–1910 (2004). 15364901 10.1101/gr.2722704 PMC524413

[69] L. Yang , Argonaute 1 regulates the fate of germline stem cells in *Drosophila*. Development **134**, 4265–4272 (2007). 17993467 10.1242/dev.009159

[70] K.-I. Nonomura , A germ cell-specific gene of the ARGONAUTE family is essential for the progression of premeiotic mitosis and meiosis during sporogenesis in rice. Plant Cell **19**, 2583–2594 (2007). 17675402 10.1105/tpc.107.053199 PMC2002623

[71] M. Singh , Production of viable gametes without meiosis in maize deficient for an ARGONAUTE protein[W]. Plant Cell **23**, 443–458 (2011). 21325139 10.1105/tpc.110.079020 PMC3077773

[72] C. Girard , RNAi-related Dicer and Argonaute proteins play critical roles for meiocyte formation, chromosome-axes lengths and crossover patterning in the fungus *Sordaria macrospora*. Front. Cell Dev. Biol. **9**, 684108 (2021). 34262901 10.3389/fcell.2021.684108 PMC8274715

[73] G. Martínez, K. Panda, C. Köhler, R. K. Slotkin, Silencing in sperm cells is directed by RNA movement from the surrounding nurse cell. Nat. Plants **2**, 16030 (2016). 27249563 10.1038/nplants.2016.30

[74] J. Brennecke , Discrete small RNA-generating loci as master regulators of transposon activity in *Drosophila*. Cell **128**, 1089–1103 (2007). 17346786 10.1016/j.cell.2007.01.043

[75] M. A. Carmell , MIWI2 is essential for spermatogenesis and repression of transposons in the mouse male germline. Dev. Cell **12**, 503–514 (2007). 17395546 10.1016/j.devcel.2007.03.001

[76] M. H. Fabry , Maternally inherited piRNAs direct transient heterochromatin formation at active transposons during early *Drosophila* embryogenesis. eLife **10**, e68573 (2021). 34236313 10.7554/eLife.68573 PMC8352587

[77] E. Dinatale, R. J. Craig, C. Martinho, H.-G. Drost, S. M. Coelho, Characterization of the transposable element landscape shaping the *Ectocarpus* genome. Genome Biol. **26**, 320 (2025). 41023682 10.1186/s13059-025-03742-z PMC12477816

[78] A. Martin-Merchan, B. Moro, A. Bouet, N. G. Bologna, Domain organization, expression, subcellular localization, and biological roles of ARGONAUTE proteins in *Arabidopsis thaliana*. J. Exp. Bot. 74, erad030 (2023). 10.1093/jxb/erad030 36722331

[79] D. C. Wallis, C. M. Phillips, RG motifs promote piRNA-mediated gene silencing in *C. elegans*. Nucleic Acids Res. **53**, gkaf1331 (2025). 41414669 10.1093/nar/gkaf1331 PMC12714567

[80] Y. Xu , The N-terminal extension of Arabidopsis ARGONAUTE 1 is essential for microRNA activities. PLoS Genet. **19**, e1010450 (2023). 36888599 10.1371/journal.pgen.1010450 PMC9994745

[81] R. Yashiro , Piwi nuclear localization and its regulatory mechanism in *Drosophila* ovarian somatic cells. Cell Rep. **23**, 3647–3657 (2018). 29925005 10.1016/j.celrep.2018.05.051

[82] P. Liu , 3D chromatin maps of a brown alga reveal U/V sex chromosome spatial organization. Nat. Commun. **15**, 9590 (2024). 39505852 10.1038/s41467-024-53453-5 PMC11541908

[83] A. Montecinos , Species delimitation and phylogeographic analyses in the *Ectocarpus* subgroup siliculosi (Ectocarpales, Phaeophyceae). J. Phycol. **53**, 17–31 (2017). 27454456 10.1111/jpy.12452

[84] S. M. Coelho , How to cultivate *Ectocarpus*. Cold Spring Harb. Protoc. **2012**, 258–261 (2012). 22301662 10.1101/pdb.prot067934

[85] S. M. Coelho , Genetic crosses between *Ectocarpus* strains. Cold Spring Harb. Protoc. **2012**, 262–265 (2012). 22301663 10.1101/pdb.prot067942

[86] G. G. Cossard , Selection drives convergent gene expression changes during transitions to co-sexuality in haploid sexual systems. Nat. Ecol. Evol. **6**, 579–589 (2022). 35314785 10.1038/s41559-022-01692-4 PMC9085613

[87] M. Hoshino , Parallel loss of sexual reproduction in field populations of a brown alga sheds light on the mechanisms underlying the emergence of asexuality. Nat. Ecol. Evol. **8**, 1916–1932 (2024). 39152327 10.1038/s41559-024-02490-w PMC11461277

[88] J. S. Lotharukpong , A transcriptomic hourglass in brown algae. Nature **635**, 129–135 (2024). 39443791 10.1038/s41586-024-08059-8 PMC11540847

[89] A. M. Bolger, M. Lohse, B. Usadel, Trimmomatic: A flexible trimmer for Illumina sequence data. Bioinformatics **30**, 2114–2120 (2014). 24695404 10.1093/bioinformatics/btu170 PMC4103590

[90] R. Schmieder, R. Edwards, Quality control and preprocessing of metagenomic datasets. Bioinformatics **27**, 863–864 (2011). 21278185 10.1093/bioinformatics/btr026 PMC3051327

[91] A. Dobin , STAR: Ultrafast universal RNA-seq aligner. Bioinformatics **29**, 15–21 (2013). 23104886 10.1093/bioinformatics/bts635 PMC3530905

[92] Y. Liao, G. K. Smyth, W. Shi, Featurecounts: An efficient general purpose program for assigning sequence reads to genomic features. Bioinformatics **30**, 923–930 (2014). 24227677 10.1093/bioinformatics/btt656

[93] M. I. Love, W. Huber, S. Anders, Moderated estimation of fold change and dispersion for RNA-seq data with DESeq2. Genome Biol. **15**, 550 (2014). 25516281 10.1186/s13059-014-0550-8 PMC4302049

[94] B. Langmead, C. Trapnell, M. Pop, S. L. Salzberg, Ultrafast and memory-efficient alignment of short DNA sequences to the human genome. Genome Biol. **10**, R25 (2009). 19261174 10.1186/gb-2009-10-3-r25 PMC2690996

[95] C. Diesh , JBrowse 2: A modular genome browser with views of synteny and structural variation. Genome Biol. **24**, 74 (2023). 37069644 10.1186/s13059-023-02914-z PMC10108523

[96] S. Lopez-Gomollon, S. Y. Müller, D. C. Baulcombe, Interspecific hybridization in tomato influences endogenous viral sRNAs and alters gene expression. Genome Biol. **23**, 120 (2022). 35597968 10.1186/s13059-022-02685-z PMC9124383

[97] C. Camacho , BLAST+: Architecture and applications. BMC Bioinformatics **10**, 421 (2009). 20003500 10.1186/1471-2105-10-421 PMC2803857

[98] J. Abramson , Accurate structure prediction of biomolecular interactions with AlphaFold 3. Nature **630**, 493–500 (2024). 38718835 10.1038/s41586-024-07487-w PMC11168924

[99] S. Guindon , New algorithms and methods to estimate maximum-likelihood phylogenies: Assessing the performance of PhyML 3.0. Syst. Biol. **59**, 307–321 (2010). 20525638 10.1093/sysbio/syq010

[100] F. Lemoine , NGPhylogeny.fr: New generation phylogenetic services for non-specialists. Nucleic Acids Res. **47**, W260–W265 (2019). 31028399 10.1093/nar/gkz303 PMC6602494

[101] A. Wallmann, M. Van De Pette, Structural and evolutionary determinants of Argonaute function. bioRxiv [Preprint] (2024). https://biorxiv.org/lookup/doi/10.1101/2024.10.10.617571 (Accessed 28 April 2025). 10.1093/nar/gkaf962 PMC12477606 41020504

[102] I. Letunic, P. Bork, Interactive tree of life (iTOL) v6: Recent updates to the phylogenetic tree display and annotation tool. Nucleic Acids Res. **52**, W78–W82 (2024). 38613393 10.1093/nar/gkae268 PMC11223838

[103] V. Bukhanets , Ectocarpus small-RNA and RNA-se. NCBI SRA. https://www.ncbi.nlm.nih.gov/sra/PRJNA1294542. Deposited 22 July 2025.

