# Supplementary material for: Germline fate determination by a single ARGONAUTE protein in Ectocarpus
Source: Proc Natl Acad Sci U S A. 2026 Jan 28;123(5):e2518712123. doi: 10.1073/pnas.2518712123 (PMC12867755; doi:10.1073/pnas.2518712123)
Supplement: Supplementary file 1 — Appendix 01 (PDF) [file pnas.2518712123.sapp.pdf]

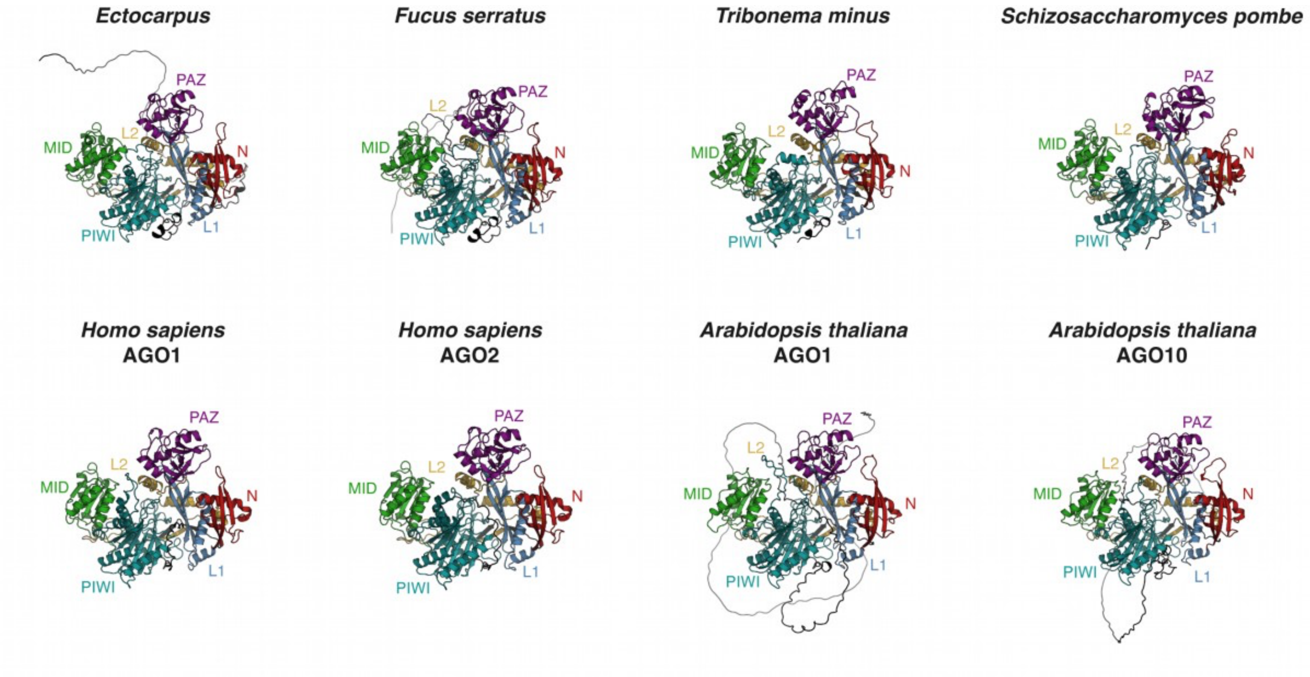

Figure S1. Eukaryotic ARGONAUTE protein structures. (A) AlphaFold3-predicted structures of representative ARGONAUTE proteins from different eukaryotes. Each structure is color-coded by domain: N-terminal (red), L1 (blue), PAZ (purple), L2 (gold), MID (green), and PIWI (teal). Unstructured N-terminal regions (IDRs) are indicated in light grey.

```
Ectocarpus AGO      MSSRG-------GGRG------GR----GRGDFRGGGRGGGGGGRGGDSY----RGGGRGGGR-G---------GGRGDFR-GGGRG---------GGRGG-GGGRFGGA
F. serratus AGO    MSDRG-------RGRGRGPRDNGR---GGRGDYRGGGRGGRG------DY----RGGGRGDFRGG---------GGRGDFRGGGGRGDFRGG---RGGRGG-GGGRFGGG
T. minus AGO       M-------------------------------------------------------------------------------------------------------------
A. thaliana AGO1  MVRKRRTDAPSEGGEGSGSREAGPVSGGGRGSQRGGFQQGGGQHQGGRGYTPQPQQGGRGGRGYGQPPQQQQQYGGPQEYQ-GRGRG----GPPHQGGRGGYGGGRGGGP
S. pombe AGO M     -------------------------------------------------------------------------------------------------------------
H. sapiens AGO1   M-------------------EAG---------------------------------------------------------------------------------------

Ectocarpus AGO      QEDARFR-NVPRVHE-----------IPI-VNVPGKPLPPP---------------APVSRTALAETPVDDIILPHRPNYGKMGKQVVVTANHYKADYNSKQLLYQYDVS
F. serratus AGO    QEDARFR-NVPRVHE-----------IPT-QMVSGKTLPRP---------------APVSRSALAETRVEDIVLPHRPNYGVNGKQVVVTANHYLADYNQTQLLYQYDVS
T. minus AGO       -------------------------------------------------------------SALRTTPVEQISFCARPGYAQKGRTTALFANHYKVDFNPAAIVQQYDFK
A. thaliana AGO1  SSGPPQRQSVPELHQATSPTYQAVSSQPTLSEVSPTQVPEPTVLAQQFEQLSVEQGAPSQAIQPIPSSSKAFKFPMRPGKGQSGKRCIVKANHFFAEL-PDKDLHHYDVT
S. pombe AGO       ----------------------------------------------------------------SYKPSSEIAL--RPGYGGLGKQITLKANFFQIISLPNETINQYHVI
H. sapiens AGO1   ---------------------------PS-GAAAGAYLPPL---------------------------QQVFQAPRRPGIGTVGKPIKLLANYFEVDI-PKIDVYHYEVD
```

N domain

```
Ectocarpus AGO      LEGFEKTALPAAKLRAIF--QKFKEQHSASSLGGIAFTYDGRSVMITARPLPFPAEGAS--FVVVFEPATEKREANNFTVILKQVAQRRLADLAVFFSGQTSQNA--YDC
F. serratus AGO    LEGFEKTALPASKLRAIF--QKFKEQHSAKSLKGIAFTYDGRSVMITASELPFGAEGQS--FVVELEPATAKREANTFTVILKKVATRQLSDLASFFKGNTTQNA--YDC
T. minus AGO       LEGIDINDMPSKKLKTIM--KAVIKQRR-ETFGDARLAFDGRSIVYASKMLPFDNEVTV--ECEIEDERSTDRPPRKYMVKLLYTAERPLTEMQAFLRGERRESA--VDV
A. thaliana AGO1  I---TPEVTSRGVNRAVM--KQLVDNYRDSHLGSRLPAYDGRKSLYTAGPLPFNSKEFRINLLDEEVGAGGQRREREFKVVIKLVARADLHHLGMFLEGKQSDAP--QEA
S. pombe AGO       VG--DGSRVPRKQSQLIWNSKEVKQYFGSSWMNS---VYDGRSMCWSKGDIADGTIKV---------NIGSESHPREIEFSIQKSSKINLHTLSQFVNSKYSSDPQVLSS
H. sapiens AGO1   I---KPDKCPRRVNREVV--EYMVQHFKPQIFGDRKPVYDGKKNIYTVTALPIGNERVD-----FEVTIPGEGKDRIFKVSIKWLAIVSWRMLHEALVSGQIPVP--LES
```

L1 linker subdomain

```
Ectocarpus AGO      ITALDISLRHAPSMKLTCVGRSFFTPDMP--SPISGGAEVWLGYYQSLRATQAGLTLNVDMSAMAFVRSMPMMDFVCELLGVRDPGQLSRGIRPYD-RRKLETALKGVNV
F. serratus AGO    ITALDISLRHAPSMALTCVGRSFYSPEMP--SPISGGAEVWLGYYQSLRATQAGLTLNVDMSAMAFVRSMPMMDFVCELLGIRDPGMLSRGIRPYD-KRKLETALKGVNV
T. minus AGO       INALDVVLRTVPTMKLVSAGRNFYMPTSD--YPISGGAEVWLGYNQSLRPGQAGTTFNADLAASAFLKVMPVMEFICQQLNCRDPRQLQQALGDMRGQKQASKAIATLAI
A. thaliana AGO1  LQVLDIVLRELPTSRYIPVGRSFYSPDIGKKQSLGDGLESWRGFYQSIRPTQMGLSLNIDMSSTAFIEANPVIQFVCDLLN-RDIS--SRPLSDAD-RVKIKKALRGVKV
S. pombe AGO       IMFLDLLLKKKPSETLFGFMHSFFTGENG--VSLGGGVEAWKGFYQSIRPNQGFMSVNVDISSSAFWRNDSLLQILMEYTDCSNVRDLTR----FD-LKRLSRKFRFLKV
H. sapiens AGO1   VQALDVAMRHLASMRYTPVGRSFFSPPEGYYHPLGGGREVWFGFHQSVRPAMWKMMLNIDVSATAFYKAQPVIEFMCEVLDIRNIDEQPKPLTDSQ-RVRFTKEIKGLKV
```

PAZ domain

```
Ectocarpus AGO      EVTHRK------SNRQYRVSALSKIGADQLTFP-DQESGRD--EIVARYFGEKY-FK-LRYPSLPCVRIGSASKHNYIPMEVCQIAQGQKVA----KLDEKQTADMIKIT
F. serratus AGO    EVTHRK------SNRQYRISAVTRQGADQTSFP-DQESGKD--LIVAHYFREKY-YP-LKYPSLPCVRVGSAAKHNYIPMEVCQIATGQRVT----KLDEKQTADMIKIT
T. minus AGO       TVNHRP------TKRTFRVSGLSRTPASGTFI--QLEGGKQ-GPSVADYFKEKYKKQGLQFPHLPCIRIGSAAKSNFLPIEVCDVAPGHRFTNAGLKLSDRQTADLIKIA
A. thaliana AGO1  EVTHRGN-----MRRKYRISGLTAVATRELTFPVDERNTQK---SVVEYFHETYGFR-IQHTQLPCLQVGNSNRPNYLPMEVCKIVEGQRYSK---RLNERQITALLKVT
S. pombe AGO       TCQHRNNVGTDLANRVYSIEGFSSKSASDSFFV-RRLNGEEQKISVAEYFLENHNVR-LQYPNLPCILVKNGA---MLPIEFCFVVKGQRYTA---KLNSDQTANMIRFA
H. sapiens AGO1   EVTHCGQ-----MKRKYRVCNVTRRPASHQTFPLQLESGQTVECTVAQYFKQKYNLQ-LKYPHLPCLQVGQEQKHTYLPLEVCNIVAGQRCIK---KLTDNQTSTMIKAT
```

L2 linker subdomain

```
Ectocarpus AGO      CQRPDVRQGAIHQQFNNINADMNKSCEQFGIRITNKQIQTQARILPPPCIQYNKAGRQQTEQPQCGSWNLRDKKMFD--NKKLVSWAVVCFTQERDLQLQGAEHFVSELV
F. serratus AGO    CQRPDVRQGAIHQQFNNINDDMNKSCQQFGIRITNKQIQTTARILPPPEIQYNKSGRQPTEKPQCGSWNLRDKKMYD--NKKLKSWAVVSFSSERDLPQHVLENFVSELV
T. minus AGO       CRRPEERRGLVHNQLGMLMGELNEHAHDFGMRVHNKAIAMTGHILPVPEIKYAN----GNERPSAGKWNMMGKKFSE--AAELACWAIICMCGPRDMQLNDVQQFAVNFV
A. thaliana AGO1  CQRPIDREKDILQTVQLNDYAKDNYAQEFGIKISTSLASVEARILPPPWLKYHESGREGTCLPQVGQWNMMNKKMIN--GGTVNNW--ICINFSRQVQDNLARTFCQELA
S. pombe AGO       VQRPFERVQQIDDFVHQMDWDTDPYLTQYGMKIQKKMLEVPARVLETPSIRYGG---DCIERPVSGRWNLRGKRFLDPPRAPIRSWAVMCFTSTRRLPMRGIENFLQTYV
H. sapiens AGO1   ARSAPDRQEEISRLMKNASYNLDPYIQEFGIKVKDDMTEVTGRVLPAPILQYGGRNR-AIATPNQGVWDMRGKQFYN--GIEIKVWAIACFAPQKQCREEVLKNFTDQLR
```

MID domain

```
Ectocarpus AGO      KVMGTHGMDVSPEARRPPILMADSVAANNQRVDDATYARNALTAARD-AARKKFKVDCQLILVPKPTQDSKDYGEIKLASDTVLGIPSQCVLLKHVHTAKIQYLANLCLK
F. serratus AGO    KVMGIHGMEVSPEARRPPLLNAEQAPGNRQRVDEVTFARNALMAARD-AARNAFKSDCQLILVPKSTTDSKDYGEIKLASDTALGIPSQCVLLKHVQTAKIQYLANLCLK
T. minus AGO       SNAKKIGMKVSME--RPPIMM---------MFDERLSIPDAMQSAFDQAAKAGRRPKPQLLLCIMPVKPFPDYPQVKLVGDTQLGVATQLLWVKHARDGKPTVLANVLLK
A. thaliana AGO1  QMCYVSGMAFNPEPVLPPV------SARPEQVEKVLKTRYHDATS-----KLSQGKEIDLLIVILPDNNGSLYGDLKRICETELGIVSQCCLTKHVFKMSKQYMANVALK
S. pombe AGO       QTLTSLGINFVMK--KPPVLY----ADIRGSVEELCITLYKKAEQ-------VGNAPPDYLFFILDKNSPEPYGSIKRVCNTMLGVPSQCAISKHILQSKPQYCANLGMK
H. sapiens AGO1   KISKDAGMPIQGQPCFCKY------AQGADSVEPMFRHLKNTYSG------------LQLIIVILPGKT-PVYAEVKRVGDTLLGMATQCVQVKNVVKTSPQTLSNLCLK

Ectocarpus AGO      INAKLGGRNAVPRDKL----PFVQDAPTIVFGADVNHPGAGNVSKPSIAAVVASMD-RWVSRHGSCVAVQEHRKEVIQDL-------------ASMVKNLLISFYRVNNA
F. serratus AGO    INAKLGGRNAVPKDKL----PFVQDAPTIVFGADVNHPGAGNVSKPSIAAVVASMD-RWVSRHGSCVAVQEHRKEVIQDL-------------AAMVKNLLISFYRVNNS
T. minus AGO       VNAKIGGRNAA--DCT----YQRTTCVIVCVCADVHHASPGS-SRPSIAAVVASLD-NQFVRHAACVRVQGPRKEIIEDL-------------AGMVRKLIMLFHSINRV
A. thaliana AGO1  INVKVGGRNTVLVDALSRRIPLVSDRPTIIFGADVTHPHPGEDSSPSIAAVVASQDWPEITKYAGLVCAQAHRQELIQDLFKEWKDPQKGVVTGGMIKELLIAFRRSTGH
S. pombe AGO       INVKVGGINCSLIPKS----NPLGNVPTLILGGDVYHPGVGA-TGVSIASIVASVD-LNGCKYTAVSRSQPRHQEVIEGM-------------KDIVVYLLQGFRAMTKQ
H. sapiens AGO1   INVKLGGINNILVPHQ---RSAVFQQPVIFLGADVTHPPAGDGKKPSITAVVGSMD-AHPSRYCATVRVQRPRQEIIEDL-------------SYMVRELLIQFYKSTRF
```

PIWI domain

```
Ectocarpus AGO      KPARIIFFRDGVSEGQFREVLRYEVRAIEQACAALEVGYRPTITFIVVQKRHHTRLFQPNRDDQ---DKSGNVFPGTVVETGICHPMEWDFYLMSHGGLQGTSRPAKYHV
F. serratus AGO    KPSRIIFFRDGVSEGQFREVLRFEVRAIEQACASLESGYRPSITFIVVQKRHHTRLFQPRREDQ---DKSGNVFPGTVVETGICHPMEWDFYLMSHGGLQGTSRPAKYHV
T. minus AGO       YPQKVVFLRDGVSEGQFRQVLKLEVRAIEEACRSISSDYRPLITFLTCQKRHHTRIYAVNAQDQ---DQSGNTQAGTVVDTAICHPTEFDFFLCSHGGLQGTSRPTKYSV
A. thaliana AGO1  KPLRIIFYRDGVSEGQFYQVLLYELDAIRKACASLEAGYQPPVTFVVVQKRHHTRLFAQNHNDRHSVDRSGNILPGTVVDSKICHPTEFDFYLCSHAGIQGTSRPAHYHV
S. pombe AGO       QPQRIIYFRDGTSEGQFLSVINDELSQIKEACHSLSPKYNPKILVCTTQKRHHARFFIKNKSDG---DRNGNPLPGTIIEKHVTHPYQYDFYLISHPSLQGVSVPVHYTV
H. sapiens AGO1   KPTRIIFYRDGVPEGQLPQILHYELLAIRDACIKLEKDYQPGITYIVVQKRHHTRLFCADKNER--IGKSGNIPAGTTVDTNITHPFEFDFYLCSHAGIQGTSRPSHYYV

Ectocarpus AGO      LWDENAFDSD SLQLLCYHLCFMYCRCTRSVSIPPAVYYAHLVAFRAQFFVN--VGDDSSSESSSV-----VQGRDGG---AREIDWARCFSSVHQSLTNVMYFV
F. serratus AGO    LWDENKFDSD SLQLLCYHLCFMYCRCTRSVSIPPAVYYAHLVAFRAQFFVN--VGDESSSDNSSV-----VHGKEKE---AKEIDWARCFSSVHSGLTNVMYFV
T. minus AGO       LWDEMNFSPD QIQKLIYELCYLQARCTRSVSIPPAVYYAHLAAFRAQHFVQ-----DMDIESQSA-----ISGRSGGSGSAISIDWSERFTNVHEDLTNVMYFV
A. thaliana AGO1  LWDENNFTAD GLQSLTNNLCYTYARCTRSVSIVPPAYYAHLAAFRARFYMEPETSDSGSMASGSMARGGGMAGRSTRGP--NVNAAVRPLPALKENVKRVMFYC
S. pombe AGO       LHDEIQMPPD QFQTLCYNLCYVYARATSAVSLVPPVYYAHLVSNLARYQDV--TADDTFVETSEA-----SMDQE-----------VKPLLALSSKLKTKMWYM
H. sapiens AGO1   LWDDNRFTAD ELQILTYQLCHTYVRCTRSVSIPAPAYYARLVAFRARYHLV---DKEHDSGEGSH-----ISGQSNG---RDPQALAKAV-QVHQDTLRTMYFA
```

Figure S2. Multiple sequence alignment between different brown algal, *A. thaliana*, *S. pombe* and *H. sapiens* ARGONAUTE proteins.

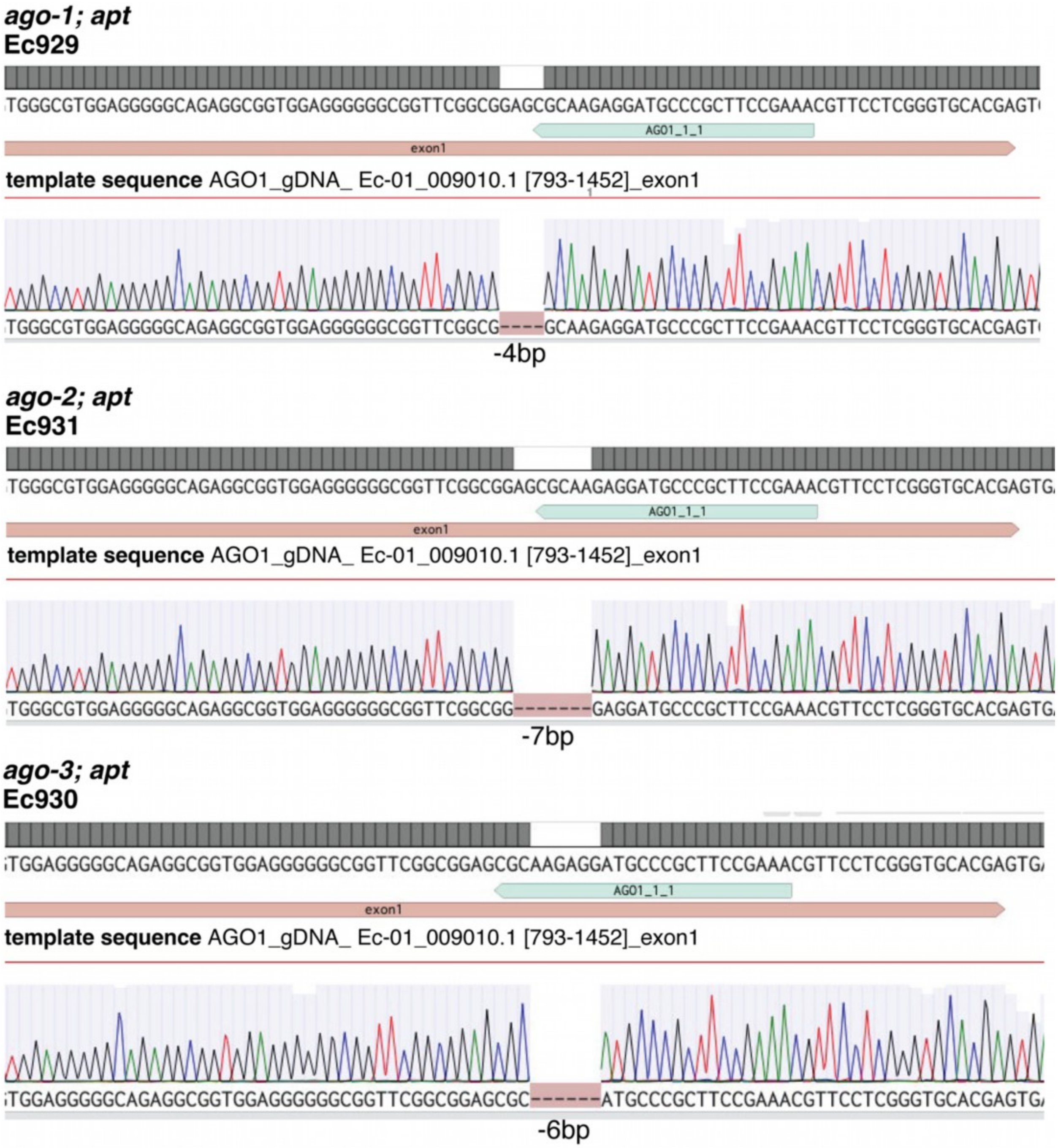

Figure S3. Sanger sequencing chromatograms of the different *ago* mutant strains used in this study. Chromatogram sequences are aligned to the reference wild type *AGO (Ec-01_009010*) sequence of *Ectocarpus*.

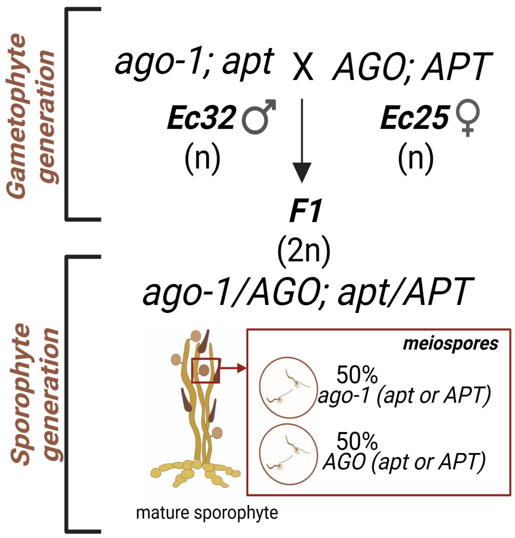

Figure S4. Crossing scheme used to test the involvement of sporophytic AGO in meiospore viability (non-cell-autonomous effect). *ago-1; apt* haploid male gametophytes generated in the Ec32 strain were isolated and crossed with wild type (*AGO; APT*) haploid female gametophytes in the Ec25 strain to obtain a heterozygous diploid sporophyte (*ago-1/AGO; apt/APT* sporophyte) with one functional *AGO* copy. The theoretical expected *ago-1* and *AGO* Mendelian allele segregation in meiospores is depicted in the red box.

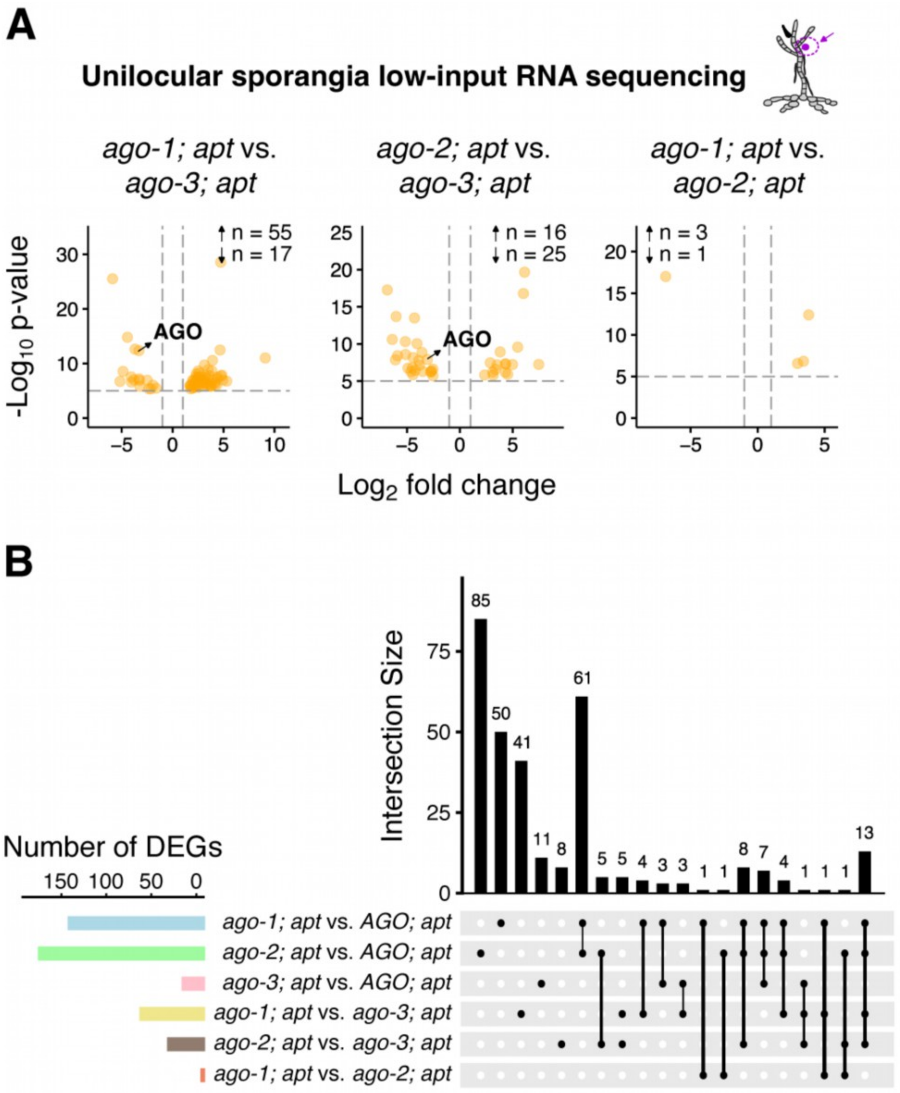

Figure S5. Transcriptomic analysis of *ago* mutants using bulk partheno-sporophyte and low-input unilocular sporangia mRNA sequencing. (A) DEGs identified by low-input mRNA sequencing of micro-dissected unilocular sporangia. Strain comparisons are indicated above each plot. *n* refers to the number of upregulated (↑) and downregulated (↓) DEGs in each comparison. Only DEGs are shown. (B) UpSet plot showing the overlap of DEGs from the comparisons in panel C. Detailed comparisons between strains and corresponding DEG lists can be found in Tables S6 and S7.
